# Inter-α-inhibitor heavy chain-1 has an integrin-like 3D structure mediating immune regulatory activities and matrix stabilization during ovulation

**DOI:** 10.1074/jbc.RA119.011916

**Published:** 2020-03-06

**Authors:** David C. Briggs, Alexander W. W. Langford-Smith, Holly L. Birchenough, Thomas A. Jowitt, Cay M. Kielty, Jan J. Enghild, Clair Baldock, Caroline M. Milner, Anthony J. Day

**Affiliations:** ‡ Wellcome Centre for Cell-Matrix Research, School of Biological Sciences, Faculty of Biology, Medicine & Health, University of Manchester, Manchester Academic Health Science Centre, Manchester M13 9PT, United Kingdom; § Department of Molecular Biology & Genetics, University of Aarhus, 8000 Aarhus C, Denmark; ¶ Division of Cell-Matrix Biology & Regenerative Medicine, School of Biological Sciences, Faculty of Biology, Medicine & Health, University of Manchester, Manchester Academic Health Science Centre, Manchester M13 9PT, United Kingdom; ‖ Lydia Becker Institute of Immunology and Inflammation, Faculty of Biology, Medicine & Health, University of Manchester, Manchester M13 9PL, United Kingdom

**Keywords:** extracellular matrix, hyaluronan, inflammation, innate immunity, protein stability, proteoglycan, reproduction, X-ray crystallography, small-angle X-ray scattering (SAXS), Inter-α-inhibitor Heavy Chain

## Abstract

Inter-α-inhibitor is a proteoglycan essential for mammalian reproduction and also plays a less well-characterized role in inflammation. It comprises two homologous “heavy chains” (HC1 and HC2) covalently attached to chondroitin sulfate on the bikunin core protein. Before ovulation, HCs are transferred onto the polysaccharide hyaluronan (HA) to form covalent HC·HA complexes, thereby stabilizing an extracellular matrix around the oocyte required for fertilization. Additionally, such complexes form during inflammatory processes and mediate leukocyte adhesion in the synovial fluids of arthritis patients and protect against sepsis. Here using X-ray crystallography, we show that human HC1 has a structure similar to integrin β-chains, with a von Willebrand factor A domain containing a functional metal ion-dependent adhesion site (MIDAS) and an associated hybrid domain. A comparison of the WT protein and a variant with an impaired MIDAS (but otherwise structurally identical) by small-angle X-ray scattering and analytical ultracentrifugation revealed that HC1 self-associates in a cation-dependent manner, providing a mechanism for HC·HA cross-linking and matrix stabilization. Surprisingly, unlike integrins, HC1 interacted with RGD-containing ligands, such as fibronectin, vitronectin, and the latency-associated peptides of transforming growth factor β, in a MIDAS/cation-independent manner. However, HC1 utilizes its MIDAS motif to bind to and inhibit the cleavage of complement C3, and small-angle X-ray scattering–based modeling indicates that this occurs through the inhibition of the alternative pathway C3 convertase. These findings provide detailed structural and functional insights into HC1 as a regulator of innate immunity and further elucidate the role of HC·HA complexes in inflammation and ovulation.

## Introduction

Inter-α-inhibitor (IαI)^3^ is a plasma proteoglycan composed of two homologous “heavy chains” (HC1 and HC2) covalently attached to chondroitin sulfate (CS) on the bikunin core protein (1; see Fig. S1*A*). IαI plays a critical role in mammalian reproductive biology such that female mice with the bikunin gene deleted, and consequently lacking IαI as well as the related pre-α-inhibitor (PαI), are infertile (2, 3). This is due to the impaired formation of the cumulus extracellular matrix that normally drives the expansion of the cumulus–oocyte complex (COC). This elastic matrix (4) protects the oocyte during the expulsion of the COC from the ovarian follicle and also provides a large surface area facilitating sperm capture *in vivo* (5, 6). The cumulus matrix is rich in the nonsulfated glycosaminoglycan hyaluronan (HA), where this high-molecular-weight polysaccharide becomes modified by the covalent attachment of HC1 and HC2 from IαI and HC3 from PαI (7). TSG-6 plays a catalytic role in transferring HCs from the CS chains of IαI and PαI onto HA to form HC·HA complexes (8, 9), where this is essential for female fertility (10–13). As well as being expressed by cumulus cells during ovulation, TSG-6 is also produced in the context of inflammation, where it mediates the formation of HC·HAs (14), *e.g.* when IαI/PαI leaks into tissues from the blood circulation (reviewed in Ref. 9).

In IαI, HC1, and HC2 (the protein products of the *ITIH1* and *ITIH2* genes) are covalently bound via ester bonds linking their C termini to GalNAc sugars within the CS chain (15, 16). The two HCs are attached to sugars one or two disaccharides apart, with HC2 positioned closer to bikunin than HC1 (17, 18). HC1 and HC2 are ∼80 kDa in size and share ∼39% sequence identity. They are synthesized with C-terminal pro-domains (of 239 and 244 amino acid residues, respectively) that are removed when the HCs are covalently attached to the bikunin CS chain (19, 20). HC3 (ITIH3; 54% identical to HC1) can also link to the bikunin CS proteoglycan (Fig. S1*A*) to form PαI (1, 21), and there is evidence that the related HC5, and likely HC6, can also become attached to CS in this way (9, 22). All HCs are predicted to contain a single von Willebrand factor type A (vWFA) domain; however, there are no structural data yet available for heavy chains.

The covalent attachment of HCs to HA changes the physical properties of this ubiquitous glycosaminoglycan. For example in synovial fluid from rheumatoid arthritis patients, where on average three to five HCs are attached to an HA chain of ∼2 MDa, the polysaccharide is more aggregated compared with unmodified HA (23); this has been attributed to cross-linking of HC·HA complexes via interactions between HCs based on their apparent associations visualized by EM. Given that HC1, HC2, and HC3 can all be transferred onto HA during arthritis (24), such cross-linking could potentially be mediated by homotypic and/or heterotypic HC–HC interactions; however, currently there are no biophysical data to support this. Irrespective of the mechanism, the formation of HC·HA in arthritic joints enhances the binding of HA to its major cell surface receptor, CD44, on leukocytes (25). However, it is unknown whether this, or indeed the altered hydrodynamic properties of the modified HA (26), are part of a protective process or contribute to arthritis pathology (9). In this regard, HC·HA complexes from human amniotic membrane, which are reported to contain only HC1 (27), are potently anti-fibrotic and anti-inflammatory (9, 28); HC·HAs also protect against endotoxic shock and sepsis (14, 29, 30). However, the role of HCs (including HC1) has not been determined in these processes.

IαI has been implicated as a regulator of innate immunity having been shown to be an inhibitor of the complement system, affecting the alternative, classical, and lectin activation pathways (31–33). The inhibition of the alternative and classical pathways of complement is thought to be dependent on HCs rather than bikunin (32, 33); however, most of the available data were generated using IαI, and the HC-mediated mechanisms have not been determined. In the case of the alternative pathway, IαI was found to inhibit the factor D–mediated cleavage of factor B (FB) to Bb, which occurs during the formation of the C3 convertase (C3bBb).

IαI has been found to bind to vitronectin (31), a multifunctional plasma and matrix protein that, as a well as being a regulator of complement system terminal pathway, also mediates binding to α_V_ integrins (34). Vitronectin's integrin-binding activity has an important role in epithelial repair in the context of lung homeostasis, and the adhesion and migration of epithelial cells was promoted by its interaction with IαI (31); moreover, IαI-deficient mice had impaired recovery in experimental lung injury. The association between IαI and vitronectin is reported to be of high affinity and inhibited by RGD peptides, implicating IαI's vWFA domain in the interaction.

To explore and better explain the functions of HCs, we undertook structural and biophysical characterization of the prototypical heavy chain, HC1. Here we present the crystal structure of HC1 and reveal that HC1 can form metal ion-dependent homodimers, which require a functional metal ion-dependent adhesion site (MIDAS) motif within its vWFA domain. We also show that the MIDAS is important in HC1-mediated inhibition of the alternative pathway C3 convertase via its interaction with C3 and demonstrate that HC1 can interact with vitronectin and other novel ligands (*e.g.* fibronectin and small latent complexes of transforming growth factor β (TGFβ)) in a noncanonical MIDAS-independent manner.

## Results

### Human HC1 has an integrin-like structure

**Figure 1.**
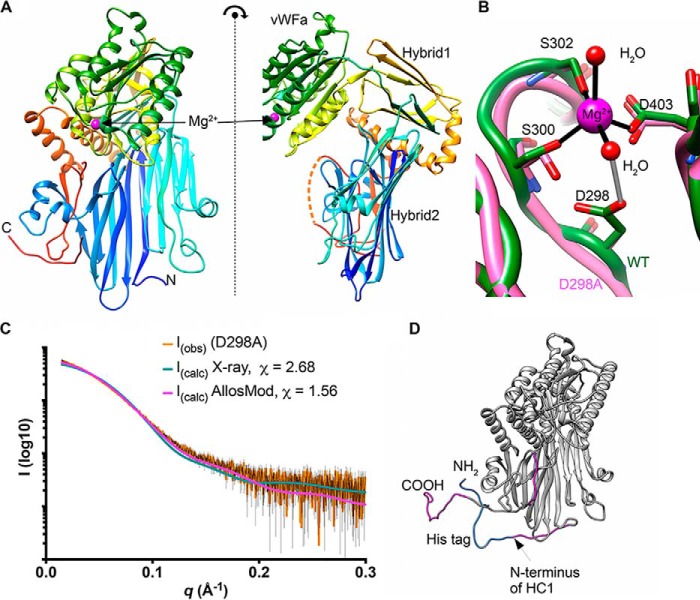
**The crystal structure of HC1.** *A*, orthogonal views of the structure of rHC1, colored from the N terminus (*blue*) to C terminus (*red*), where domains and the bound Mg^2+^ ion are labeled; the *dotted orange line* denotes residues 631–638, which are not visible in the crystal structure. *B*, close-up of the MIDAS site, showing metal coordination (*black*) and an important hydrogen bond (*gray*). The WT structure is shown in *green*, and the D298A structure (which lacks the Mg^2+^ ion) is in *pink. C*, raw SAXS data (*orange* with *black error bars*) of a rHC1 monomer (D298A) and back-calculated scattering curves based on the crystal structure of rHC1 alone or the crystal structure with the unstructured/flexible regions modeled in using AllosMod. *D*, AllosMod model of rHC1 with the N-terminal histidine tag (*blue*) and residues 35–44, 631–636, and 653–672 (*pink*) modeled based on SAXS restraints.

Crystal structures were obtained for the WT recombinant HC1 (rHC1), encompassing the entire 638-residue mature protein sequence, and for the corresponding D298A single-site mutant, at 2.34 and 2.20 Å resolution, respectively (Table 1). This revealed that heavy chains are composed of three distinct domains (Fig. 1*A* and Fig. S1*B*); these domains include a vWFA domain (residues 288–477), which is inserted into a loop in an integrin-like hybrid domain (termed here HC–Hybrid1) composed of residues 266–287 and 478–543. These two domains sit atop a large, novel, 16-stranded β-sandwich, composed of residues 45–265 and 601–652, along with 3 α-helices (residues 544–600), which together we call the HC–Hybrid2 domain (Fig. 1*A*).

**Table 1:** **Data collection and refinement statistics for rHC1**

	WT	D298A
Protein Data Bank code	6FPY	6FPZ
Wavelength	0.92 Å	0.92 Å
Resolution range*^a^*	71–2.3 (2.4–2.3)	56.8–2.2 (2.3–2.2)
Space group	P 42	P 42
Unit cell	158.8, 158.8, 65.4, 90, 90, 90	159.7, 159.7, 65.79, 90, 90, 90
Total reflections	455,794 (45,882)	312,045 (26,776)
Unique reflections	69,109 (6862)	83,837 (8233)
Multiplicity	6.6 (6.7)	3.7 (3.3)
Completeness (%)	91.94 (87.58)	94.27 (88.13)
Mean *I*/σ*I*	8.02 (1.88)	10.93 (2.05)
Wilson B-factor	37.46	34.70
*R*_merge_	0.1432 (0.8002)	0.06619 (0.4731)
*R*_meas_	0.1554 (0.8681)	0.07734 (0.5596)
*R*_pim_	0.05991 (0.3337)	0.03928 (0.2917)
CC_½_	0.994 (0.523)	0.995 (0.478)
CC*	0.998 (0.829)	0.999 (0.804)
Reflections used in refinement	63,610 (6027)	79,667 (7405)
Reflections used for *R*_free_	3194 (306)	3983 (347)
*R*_work_	0.2317 (0.3810)	0.2174 (0.3701)
*R*_free_	0.2605 (0.4195)	0.2475 (0.3641)
CC_work_	0.953 (0.726)	0.954 (0.730)
CC_free_	0.946 (0.696)	0.948 (0.768)
Number of non-hydrogen atoms	9702	10,106
Macromolecules	9319	9335
Ligands	38	58
Solvent	345	713
Protein residues	1201	1196
RMSD		
Bonds	0.004	0.003
Angles	1.03	0.92
Ramachandran (%)		
Favored	97.65	98.15
Allowed	2.27	1.85
Outliers	0.08	0.00
Rotamer outliers (%)	1.20	1.00
Clashscore	1.78	1.71
Average B-factor	49.67	45.75
Macromolecules	49.92	45.76
Ligands	62.82	71.64
Solvent	41.55	43.54
Number of TLS groups	8	6

*^a^* Statistics for the highest-resolution shell are shown in parentheses.

The construct-derived His_6_ tag and residues 35–44, 631–638, and 653–672 of HC1, which were clearly present in the protein preparation as determined by MS, were not visible in the electron density and are therefore assumed to be unstructured or highly conformationally labile. This includes the native C terminus of HC1, which is covalently attached to CS in IαI and to HA in the context of HC·HA complexes. These missing residues were modeled using small angle X-ray scattering (SAXS) data for (monomeric) D298A as a restraint target (Fig. 1, *C* and *D*); as can be seen, the AllosMod model fits better than the crystal structure alone to the experimental SAXS curve, with χ values of 1.56 and 2.68, respectively.

Despite low sequence identities (17 and 15%, respectively), the HC1 vWFA domain is structurally most similar to the vWFA domains from capillary morphogenesis protein 2 (CMG2 (35)) and tumor endothelial marker-8 (TEM8 (36)), with PDBeFold *Q* scores of 0.56 and 0.52, respectively. These are both transmembrane proteins that serve as functional receptors for the anthrax toxin (37). HC1 also shows significant structural similarity to the vWFA domains of various integrin I domains, with the highest *Q* score (0.50) for integrin α_M_ (also known as CD11b or as complement receptor type 3 (CR3)), and the vWFA domain of complement FB (*Q* score 0.37); FB and integrin α_M_ are C3-binding proteins, with roles in complement activation/amplification and complement-mediated phagocytosis, respectively (38). From the structure of WT rHC1, it is apparent that its vWFA domain contains a MIDAS motif (Fig. 1*B*), where residues Asp^298^, Ser^300^, Ser^302^, and Asp^403^ chelate a magnesium ion, the identity of which can be inferred from the trigonal bipyramid coordination geometry, bond distances, and refined atomic displacement parameters. The D298A mutant has no bound Mg^2+^ ion but is otherwise essentially identical to the WT structure, with a RMSD between the two most similar chains of 0.24 Å over 598 C-α atoms.

**Figure 2.**
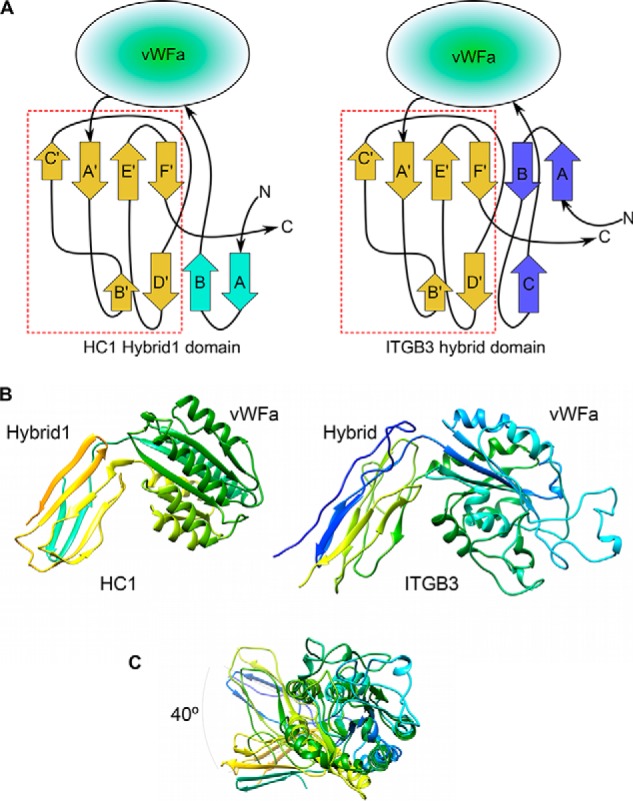
**Integrin-like arrangement of vWFA and Hydrid1 domains in HC1 structure.** *A*, topologies of the HC–Hybrid1 domain from HC1 (*left panel*) and the hybrid domain from human integrin β3 chain (ITGB3) (*right panel*); the arrangements of β-strands in the sequences following the vWFA domains are essentially identical (*dashed red box*). *B*, side-by-side views of the “hybrid” and vWFA domain pairs of HC1 (*left panel*) and ITGB3 (*right panel*). *C*, the hybrid domains are displaced by ∼40° when the vWFA domains of HC1 and ITGB3 are superimposed.

The HC–Hybrid1 domain of HC1 is composed of two four-stranded β-sheets, where two of the β-strands are formed from amino acid residues before the vWFA domain and the remaining six β-strands are from sequence after it; these regions (residues 266–287 and 478–543, respectively) are connected by a disulfide bond between Cys^268^ and Cys^540^ (39). This arrangement of the HC–Hybrid1 and vWFA domains is highly reminiscent of integrin β-chains, as illustrated in Fig. 2 for a comparison of rHC1 with ITGB3. Here the topologies of the β-strands in the hybrid domains are similar (Fig. 2*A*), as are the relative positions of the two domains (Fig. 2*B*), although being somewhat differently oriented (Fig. 2*C*). Moreover, the HC–Hybrid1 domain, which is a variant of the fibronectin type III fold, has its closest structural match in mammalian extracellular proteins to the third fibronectin type III domain from integrin IGTB4 (40), with a RMSD between the structures of 1.55 Å.

### rHC1 forms MIDAS and metal-ion dependent homodimers

**Figure 3.**
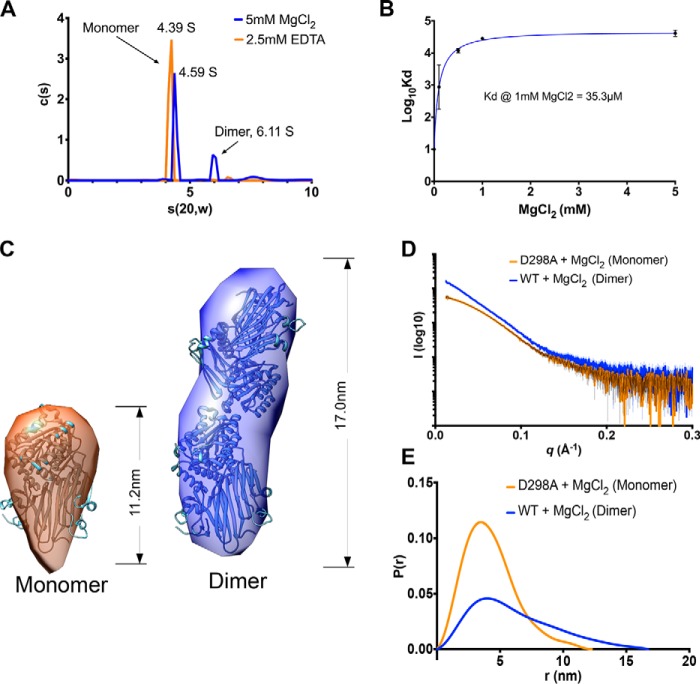
**HC1 forms metal ion-dependent dimers.** *A*, a plot of sedimentation coefficient distributions (*c*(*s*)) *versus* s(apparent)) for WT rHC1 derived from velocity AUC analysis. In the presence of 2.5 mm EDTA (*orange*) 93% of the rHC1 protein is in a monomeric state (*s*_(20,w)_ = 4.39 S), and there is no detectable dimer present. In 5 mm MgCl_2_ (*blue*), 64% of the protein is monomeric (s_(20,w)_ = 4.59 S), and 21% of material is dimeric (*s*_(20,w)_ = 6.11 S). *B*, plot of Log_10_*K_D_ versus* MgCl_2_ concentration, derived from equilibrium AUC measurements. At 0 mm MgCl_2_ (achieved by conducting the experiment in 2.5 mm EDTA), no dimerization was detected. Maximal binding affinity (for self-association of the rHC1 dimer) was reached at ∼1 mm MgCl_2_, *i.e.* close to the concentration of free Mg^2+^ ions in plasma. *C*, *ab initio* SAXS models for the HC1 monomer (*left panel*) and dimer (*right panel*) where the HC1 structure has been modeled into the SAXS envelopes. *D* and *E*, buffer-subtracted SAXS scattering curves for HC1 D298 monomer (*orange*) and WT dimer (*blue*) (*D*) and their derived *P*(*r*) *versus* distance plots (*E*), consistent with WT HC1 forming an elongated Mg^2+^-dependent dimer and the MIDAS site mutant (D298A) being monomeric.

As described above the structure of rHC1 has many similarities to integrins. This includes the presence of a vWFA domain containing a MIDAS motif known in integrins to be responsible for mediating cation-dependent ligand interactions (41, 42). During size-exclusion chromatography of rHC1, a small amount of dimer was observed when metal ions were included in the buffer. Thus, we explored this further with both analytical ultracentrifugation (AUC) and SAXS. Velocity AUC revealed that in the presence of magnesium, WT rHC1, while mostly monomeric, formed dimers (Fig. 3*A*), with sedimentation coefficients (*s*_(20,w)_) of 4.59 and 6.11 S, respectively (Table 2). Equilibrium AUC conducted at a range of magnesium ion concentrations (Fig. S2) showed that the HC1–HC1 interaction was indeed Mg^2+^-dependent, with a *K_D_* of 35.3 μm at 1 mm MgCl_2_ (Fig. 3*B*). High-throughput SAXS screening, generating *D*_max_ (maximum dimension) values, was used as a way of determining the oligomerization state of the WT and D298A proteins in different metal ion conditions (Table 2). Magnesium chloride and manganese chloride supported dimerization of WT rHC1, whereas in calcium chloride and EDTA the protein was monomeric; the D298A mutant did not form Mg^2+^- or Mn^2+^-dependent dimers. Therefore, we concluded that the dimerization activity of rHC1 requires a correctly formed and metal ion–occupied MIDAS accommodating either a magnesium or manganese ion.

**Table 2:** **Biophysical analysis of rHC1 dimerization** Radius of gyration (*R*_g_), maximum dimension (*D*_max_), approximate molecular weight (Mwt), and sedimentation coefficient (*s*_(20,w)_) values were derived from SAXS and AUC data for WT and D298A rHC1. All D298A data and WT data collected in the presence of 2.5 mm EDTA are consistent with a monomeric state. WT rHC1 with 5 mm MgCl_2_ or 5 mm MnCl_2_ is dimeric. The data from “as purified” WT rHC1 and WT rHC1 in 5 mm CaCl_2_ are consistent with a mixture of monomer and dimer; this is presumably due to trace amounts of Mg^2+^ ions present in various buffer components. The AUC data are derived from equilibrium experiments performed in triplicate at three different speeds; SAXS data are from data processed by AUTORG and with DATGNOM (*i.e.* with no imposed constraints). The molecular weight of an rHC1 monomer from intact mass spectrometry is 73,802 Da.

Protein	Metal ion	*R*_g_^SAXS^	*D*_max_^SAXS^	Mwt^SAXS^ (ratio SAXS mass/intact mass)*^a^*	*s*_(20,w)_^AUC^	*s*_(20,w)_^SAXS^
		Å	Å	*kDa*	*S*	*S*
rHC1 WT	None*^b^*	35.2	123			
	EDTA (2.5 mm)	31.8	112	78 (1.06)	4.39	4.50
	Mg^2+^ (5 mm)	49.5	170	140 (1.89)	4.59 (monomer), 6.11(dimer)	6.24
	Mn^2+^ (5 mm)	51.2	172			
	Ca^2+^ (5 mm)	38.5	135			
rHC1 D298A	None*^b^*	33.3	114			
	Mg^2+^ (5 mm)	33.3	114			
	Mn^2+^ (5 mm)	32.8	105			
	Ca^2+^ (5 mm)	32.8	115			
IαI	Mg^2+^ (5 mm)	49.2	170			

*^a^* Calculated using the method in Ref. 83.

*^b^* As purified.

SAXS data for the rHC1 monomer (D298A in MgCl_2_) and dimer (WT in MgCl_2_) were used to obtain low resolution *ab initio* solution structures (Fig. 3, *C–E*); dimensionless Kratky, SIBYLS, and Porod–Debye plots (Fig. S3) reveal the rHC1 monomer and dimer to be folded and rigid. For the monomer it was apparent that the crystal structure for rHC1 could be well-accommodated within the SAXS envelope. However, when two HC1 molecules were fitted into the envelope for the rHC1 dimer, the fitting was ambiguous; the model presented in Fig. 3*C* gives the best overall fit-to-map correlation, but other models give similar scores. This indicates that a conformational change occurs in the HC1 structure on dimerization. Moreover, small differences between the sedimentation coefficients determined by velocity AUC for the monomer species in EDTA (4.39 S) and MgCl_2_ (4.59 S) indicate that metal ion binding induces a structural change in the monomeric protein (in solution phase) prior to dimer formation; *i.e.* consistent with a recent biochemical analysis (43).

### rHC1 structure enables SAXS-based modeling of IαI

**Figure 4.**
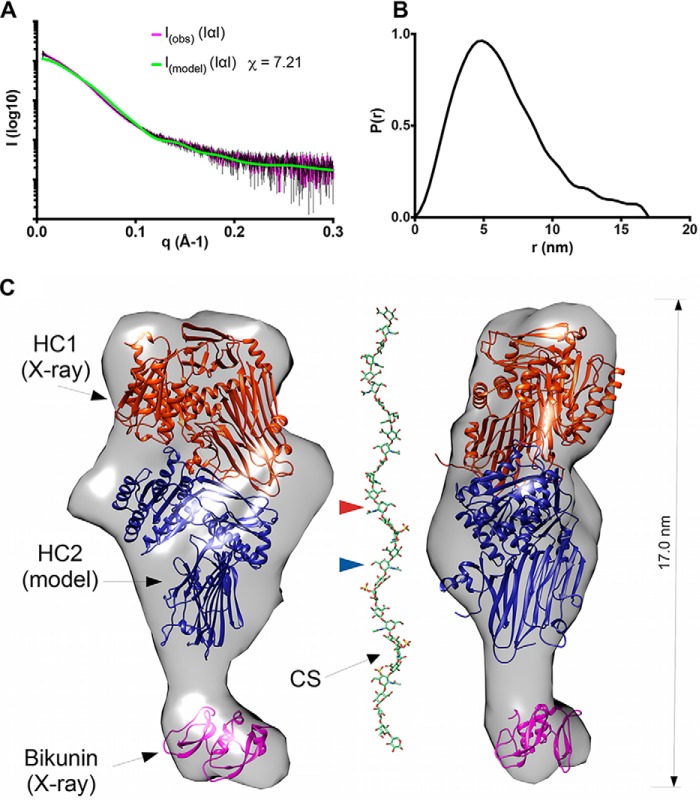
**The quaternary structure of inter-α-inhibitor.** *A*, raw SAXS data for IαI (*I*_(obs)_), in the presence of 2 mm MgCl_2_, fitted to scattering data (*pink* with *black error bars*) derived from the pseudo atomic model (*I*_(model)_) in *C* calculated using Allosmod-FoXs. *B*, *P*(*r*) *versus* distance plot showing that IαI has an elongated and asymmetric shape. *C*, orthogonal views of the SAXS envelope of IαI (*transparent gray surface*) determined *ab initio* from the SAXS scattering curve, with structures of bikunin (PDB code 1BIK; *pink*) and rHC1 (determined here; *orange*) and a threading model of HC2 (based upon the structure of HC1; *blue*), modeled in. The CS chain is shown to indicate its dimensions relative to the SAXS envelope for IαI. Here the CS chain, with a standard tetrasaccharide linker, has been modeled on the sequences determined for bikunin·CS (18) with the GLYCAN-Web GAG Builder modeling tool (82), using the median values established in the Ly *et al.* study (*a* = 2, *b* = 1, *c* = 4, and *d* = 1, corresponding to a CS chain of 26 saccharides (18)). The CS chain is attached to S10 (in the mature bikunin sequence), which is not present in the crystal structure (PDB code 1BIK) that corresponds to residues 25–134 (44). The GlcNAc moieties in the CS chain to which HC1 and HC2 are covalently attached, via their unstructured C-terminal peptides (not shown), are indicated by *arrowheads* (*orange* and *blue*, respectively).

SAXS analysis of IαI, purified from human plasma, showed it is monomeric in the presence of MgCl_2_ (Fig. 4, *A* and *B*, and Table 2). IαI, which is likely rigid (Fig. S4), has an elongated shape (Fig. 4*C*), with a *D*_max_ value of 17.0 nm, which is the same as for the HC1 dimer (Fig. 3*C*). The SAXS data (collected in 2 mm MgCl_2_) allowed the generation of an *ab initio* solution structure for IαI, enabling the determination of the likely quaternary organization of the IαI complex (Fig. 4*C*); *i.e.* by combining the SAXS data with the crystal structures of bikunin (44) and rHC1, a homology model of HC2 (based on the HC1 coordinates determined here) and the experimentally determined disulfide bond network (39). The three protein chains of IαI could be readily fitted within the SAXS envelop with the bikunin chain being accommodated in a small lobe at one end and the two HCs arranged asymmetrically in the larger lobe. This positioning would place the C-terminal peptides of HC1 and HC2 on the same face, making them close enough to take part in the observed CS conjugation (17, 18). The IαI model shown in Fig. 4*C* was used to back-calculate SAXS data, where this was found to have reasonable agreement with the experimentally derived scattering data; *i.e.* χ = 7.21 for *I*_(obs)_ *versus I*_(model)_.

### rHC1 inhibits the alternative complement pathway via C3 binding in a MIDAS-dependent manner

**Figure 5.**
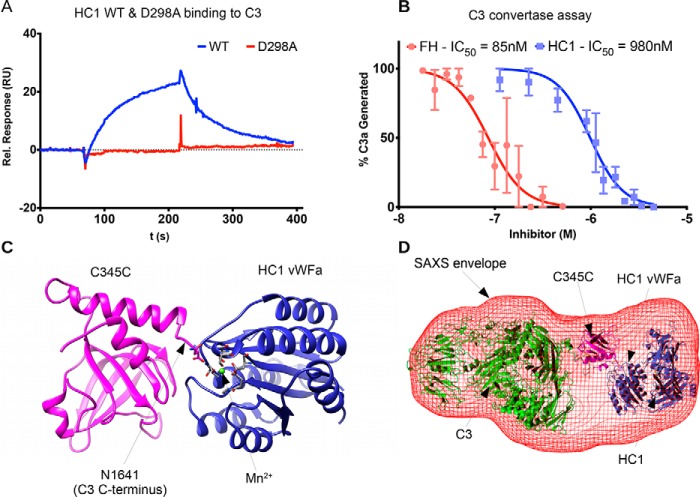
**HC1 inhibits alternative pathway C3 convertase activity through interaction with C3.** *A*, SPR analysis for the interaction of rHC1 (WT and D298A) with C3 in 2 mm MnCl_2_, where the lack of binding of the D298A mutant indicates an essential role for the MIDAS site. *B*, rHC1 proteins (WT (*blue*) and D298A) were compared with FH (*red*) in an alternative pathway C3 convertase assay, where the proteolytic release of C3a was quantified (by SDS-PAGE) as a surrogate for the conversion of C3 into C3b. The mean values (± S.D.) were derived from independent experiments performed in triplicate. The data were fitted using GraphPad Prism to derive IC_50_ values for rHC1 and FH control. Only WT rHC1 had inhibitory activity (data for D298A not shown). *C*, an *in silico* model of the C3 C-terminal C345C domain (*pink*) bound to the vWFA domain of HC1 (*blue*). Here a Mn^2+^ ion (*green*) occupies the MIDAS of HC1 (with coordinating residues shown in *stick representation*) and co-chelates the C-terminal amino acid (Asn) of C3b. *D*, an *ab initio* SAXS structure was determined for the rHC1–C3 complex (*red mesh*), where C3 and HC1 molecules, interacting as in *C*, could be accommodated.

Given that HCs are potential inhibitors of the alternative and classical complement pathways (33) and the structural similarities of rHC1 with known C3-binding proteins described above, it was investigated whether rHC1 could interact with C3 (*i.e.* the central component of the complement system). Initial buffer screening using surface plasmon resonance (SPR) revealed that rHC1 interacted with human C3 in a Mn^2+^ ion–dependent manner, which is mediated via the HC1 MIDAS motif because the D298A mutant exhibited no binding activity (Fig. 5*A* and Table 3). There was also an interaction in Mg^2+^ (albeit of lower apparent affinity), but there was no binding in the presence of Ca^2+^ or EDTA (data not shown). Full SPR analysis (in 2 mm MgCl_2_, 2 mm MnCl_2_) determined that the *K_D_* for the rHC1–C3 interaction was ∼360 nm; *i.e.* the same order of magnitude as the binding of C3 to IαI (*K_D_* = ∼660 nm; Table 3). Analysis of rHC1 in a functional assay of complement activation (compared with factor H (FH), an important inhibitor of the complement system (45)) demonstrated that the WT rHC1 protein, but not the D298A mutant, was able to dose-dependently inhibit the activity of the alternative pathway C3 convertase (C3bBb); rHC1 had an IC_50_ of 980 nm compared with the value of 85 nm obtained for FH (Fig. 5*B*).

**Table 3:** **Analysis of rHC1–ligand interactions by SPR**

Immobilized ligand	Analyte	Buffer conditions	Replicates	*K_D_* ± S.D.
				*nm*
C3	rHC1 (WT)	2 mm Mg^2+^, 2 mm Mn^2+^	3	364 ± 78
C3	rHC1 (WT)	2 mm Mn^2+^	3	473 ± 329
C3	rHC1 (D298A)	2 mm Mg^2+^, 2 mm Mn^2+^	3	NB*^a^*
C3	IαI	2 mm Mg^2+^, 2 mm Mn^2+^	3	659 ± 205
rHC1 (WT)	Vitronectin	10 mm EDTA	3	0.192 ± 0.014
rHC1 (WT)	Vitronectin	2 mm Mg^2+^, 2 mm Mn^2+^	3	0.118 ± 0.011
rHC1 (D298A)	Vitronectin	10 mm EDTA	3	0.138 ± 0.040
rHC1 (D298A)	Vitronectin	2 mm Mg^2+^, 2 mm Mn^2+^	3	0.096 ± 0.044
rHC1 (ΔvWFa)	Vitronectin	10 mm EDTA	3	0.097 ± 0.049
rHC1 (WT)	TGFβ1-LAP	2 mm Mg^2+^, 2 mm Mn^2+^	3	16.1 ± 5.5
rHC1 (D298A)	TGFβ1-LAP	2 mm Mg^2+^, 2 mm Mn^2+^	3	14.9 ± 2.7
rHC1 (WT)	TGFβ2-LAP	2 mm Mg^2+^, 2 mm Mn^2+^	3	8.8 ± 0.6
rHC1 (D298A)	TGFβ2-LAP	2 mm Mg^2+^, 2 mm Mn^2+^	3	12.9 ± 1.7
rHC1 (WT)	TGFβ3-LAP	2 mm Mg^2+^, 2 mm Mn^2+^	3	9.9 ± 1.4
rHC1 (D298A)	TGFβ3-LAP	2 mm Mg^2+^, 2 mm Mn^2+^	3	3.1 ± 0.8
rHC1 (WT)	LAP (TGFβ1)	10 mm EDTA	3	2.0 ± 0.3
rHC1 (WT)	TGFβ1	10 mm EDTA	3	NB
rHC1 (WT)	TGFβ3	10 mm EDTA	3	NB
rHC1 (WT)	LTBP1 NT1	10 mm EDTA	3	5.1 ± 0.5
rHC1 (WT)	LTBP1 NT1	2 mm Mg^2+^, 2 mm Mn^2+^	3	4.6 ± 0.2
rHC1 (WT)	LTBP1 EGF	2 mm Mg^2+^, 2 mm Mn^2+^	3	NB
rHC1 (WT)	LTBP1 CT	2 mm Mg^2+^, 2 mm Mn^2+^	3	NB
rHC1 (WT)	cFN13–14	No added M^2+^ or EDTA	3	14.0 ± 0.12
rHC1 (D298A)	cFN13–14	No added M^2+^ or EDTA	3	15.7 ± 0.18
rHC1 (ΔvWFa)	cFN13–14	No added M^2+^ or EDTA	3	16.3 ± 0.18

*^a^* NB, no binding.

The divalent cation- and MIDAS-dependent interaction of rHC1 with C3 is highly similar to the manner in which FB associates with C3b (activated C3) to form the C3 convertase; this is mediated by the vWFA domain in FB binding to the C terminus of C3/C3b via cochelation of a Mg^2+^ ion bound to FB's MIDAS motif (46). *In silico* modeling of the HC1 vWFA and the C-terminal domain (C345C) of C3/C3b (Fig. 5*C*) reveals that a MIDAS-mediated interaction is indeed feasible and consistent with a low resolution SAXS structure determined for the rHC1–C3 complex (Fig. 5*D*); although the complex is folded and globular, Porod–Debye analysis indicated that it had some flexibility (Fig. S5). Together, these data identify HC1 as a novel inhibitor of the complement alternative pathway, likely by competing for the binding of FB with C3b.

### HC1 binds to integrin ligands in a MIDAS- and vWFA-independent manner

The structural similarity of HC1 to integrin β-subunits and the finding that rHC1 dimerizes and binds to complement C3 in a metal ion- and MIDAS motif-dependent manner led us to further explore rHC1's interaction with integrin ligands. Initially the interaction with vitronectin (an RGD-containing integrin ligand) was investigated because this protein has been found to interact with IαI, and the vWFA domain has been implicated in binding (31). SPR analysis (Fig. S6) showed that rHC1 binds with high affinity to vitronectin (*K_D_* < 0.2 nm; Table 3), confirming that the HCs of IαI do mediate this interaction. However, unexpectedly, binding was independent of metal ions (Table 3) with essentially identical shaped binding curves seen for experiments in Mg^2+^/Mn^2+^ and EDTA (data not shown). Moreover, the D298A mutant and a construct where the entire vWFA domain had been removed (ΔvWFA) both bound to vitronectin with very similar affinities to the WT protein (Table 3 and Fig. S6*C*). Together, these data demonstrated definitively that the vWFA domain of HC1 does not mediate its interaction with vitronectin. Consistent with this, the interaction between WT rHC1 and vitronectin could not be competed by the GRGDPS peptide (Sigma–Aldrich), even when present at an >10,000-fold molar excess (data not shown).

The interactions of rHC1 with the small latent complexes (SLCs) of TGFβ1, TGFβ2, and TGFβ3, in which the growth factors are coupled to latency-associated peptides (LAPs), were also investigated. They were chosen because TGFβ1-LAP and TGFβ3-LAP are activated by the α_V_β_6_ and α_V_β_8_ integrins, in response to mechanical stress, in a metal ion- and MIDAS-dependent manner by binding to a RGD motif in their LAP regions (47–49). As shown in Fig. S6*D*, rHC1 could interact tightly with TGFβ1-LAP, TGFβ2-LAP (which does not contain an RGD), and TGFβ3-LAP, where the affinity (*K_D_* = ∼10 nm) was essentially identical for the WT and D298A mutant proteins (Table 3); moreover, similar binding was seen in EDTA (not shown). Together, these data demonstrate that the interactions are independent of metal ions and do not involve HC1's MIDAS motif. Additional SPR experiments (Table 3) revealed that rHC1 interacts with the LAP peptide (analyzed for the isolated LAP from TGFβ1; *K_D_* = 2 nm) but did not bind to the mature growth factor (*i.e.* TGFβ1 and TGFβ3). SLCs associate with latent TGFβ-binding proteins (LTBP) to form large latent complexes (LLCs) (50); this mediates matrix sequestration and regulates the activation of latent TGFβ. We tested whether rHC1 could bind to LTBP1 and found that it interacts with the N-terminal region (NT1), again in a metal ion-independent manner, but not with the C-terminal (CT) or EGF regions (Table 3). These experiments identify the SLC and LLC of TGFβ1, TGFβ2, and TGFβ3 as novel ligands for HC1.

We also analyzed whether rHC1 interacts with fibronectin, because this is another well-established integrin ligand. Initial SPR screening experiments with the 50-kDa and H120 fragments of fibronectin (51), corresponding to the cell- and heparin-binding regions, respectively, indicated that although both bound to rHC1 (in a metal ion-independent manner), the interaction with H120 was of considerably higher affinity than that with the 50-kDa fragment; the H120* fragment (missing the 12–13 type III repeats, but otherwise identical to H120) did not bind to rHC1. Therefore, we expressed fibronectin type III repeats 13 and 14 (denoted as cFN13–14) in *Escherichia coli*, purifying this to homogeneity (see Fig. S7 and “Experimental procedures”), and showed that this bound rHC1 in an essentially identical fashion to H120; *i.e.* localizing the high affinity HC1-binding site on fibronectin to the 13 and 14 type III domains. Full SPR analysis of the interaction between cFN13–14 and rHC1, which was carried out in the absence of metal ions or EDTA, revealed that this region of fibronectin binds with a *K_D_* of ∼15 nm to the WT, D298A, and ΔvWFA constructs (Fig. S8 and Table 3). These data demonstrate that, as for vitronectin, the MIDAS and vWFA domains of rHC1 are not involved in the interaction with fibronectin. Furthermore, rHC1 binds most tightly to a region of fibronectin that does not contain an RGD sequence. Thus, we have found that a number of integrin ligands (fibronectin, TGFβ1-LAP, TGFβ3-LAP, and vitronectin) bind to HC1 but that these interactions are not mediated by a MIDAS/RGD-binding mechanism.

## Discussion

Here we have determined the first crystal structure for a heavy chain of the IαI/ITIH family. Given the similarity of the prototypical HC1 to the five other HC proteins encoded in the human genome (32–54% sequence identity), our study defines the canonical structure for a heavy chain, allowing the modeling of other family members. In this regard, we generated a homology model of HC2 that, along with the structure for rHC1 (and bikunin), allowed us to infer the quaternary organization of IαI itself. Our SAXS-based modeling of IαI (Fig. 4) reveals that this unusual CS proteoglycan forms an elongated structure but with a compact arrangement of the three protein chains as also inferred in a recent biochemical study (43).

Unlike IαI, which we found to be monomeric, rHC1 forms a dimer in solution. Given the metal ion dependence of dimer formation (requiring Mg^2+^ or Mn^2+^; Table 2) and the lack of dimerization by the D298A mutant, the MIDAS motif within the vWFA domain clearly plays an essential role in mediating this protein–protein interaction. It is possible that an Asp or Glu side chain on one HC1 monomer could engage with the metal ion within the MIDAS of the other HC1; *e.g.* to mediate binding and effect a conformational change, thereby altering the orientation of the vWFA domain relative to the rest of the protein and leading to the dimer dimensions indicated by SAXS (Fig. 3*C*). This is reminiscent of how metal ion and ligand occupancy of an integrin MIDAS can transduce a conformational change that causes the hybrid and vWFA domains to swing away from one another during integrin activation (41, 42). The arrangement of the HC1 and HC2 vWFA domains in our IαI model (Fig. 4*C*) indicates that such interactions would be sterically precluded, explaining why IαI does not dimerize.

It is well-established that HC1, HC2, and HC3 can become covalently attached to the polysaccharide HA via transesterification reactions catalyzed by TSG-6, *e.g.* in the context of ovulation and inflammation (9). This reaction requires the presence of Ca^2+^ and Mg^2+^/Mn^2+^ ions (10) and occurs via the formation of covalent TSG-6·HC intermediates (8). There is a Ca^2+^ ion–binding site in the TSG-6 CUB_C domain, which we have shown previously to be required for TSG-6·HC formation via its initial noncovalent association with HCs (10). The finding here that an Mg^2+^or Mn^2+^ ion can be accommodated within the vWFA domain of HC1 (Fig. 1) provides strong evidence that HCs are the source of these metal ions, especially given that the D298A mutant cannot bind to CUB_C domain of TSG-6 (10). Moreover, our determination of the heavy chain structure will facilitate refinements in our understanding of the mechanisms underlying the transfer of HCs onto HA.

The dimerization of rHC1 provides direct evidence for HC–HC interactions (Fig. 3 and Table 2). Given that the C-terminal 20 amino acid residues of HC1 (which were not visible in the crystal structure) are likely to form a flexible and extended linker (as informed by SAXS-based modeling; Fig. 1*D*), this protein–protein interaction is unlikely to be adversely affected by the C terminus of HC1 being covalently attached to HA. As such, homotypic binding between HC1 molecules can contribute to the cross-linking of HC·HA complexes formed during ovulation and inflammation. However, the HC1–HC1 interaction is rather weak (*K_D_* = ∼40 μm at physiological Mg^2+^ concentrations; Fig. 3*B*), indicating that, for this heavy chain at least, binding is likely to be highly transient. As yet we do not know whether other HCs self-associate in this way or indeed the nature/affinities of heterotypic HC–HC interactions. However, it seems reasonable to propose that low-affinity binding between multiple pairs of HCs could mediate the aggregation of HC·HAs seen in synovial fluids from rheumatoid arthritis patients (23) and that this, combined with more stable interactions between HCs and pentraxin 3 (26), underpins the formation and cross-linking of the cumulus extracellular matrix during COC expansion. Furthermore, dynamic HC–HC interactions could make an important contribution to the mechanical properties of tissues; for example, they might explain the elasticity and extreme softness of the cumulus matrix (4). Certainly HC·HAs have different compositions of heavy chains in different tissue contexts (9), and it seems likely that this will engender distinct hydrodynamic and functional properties.

HC·HA complexes in inflamed synovial fluids associate more tightly with CD44 on infiltrating leukocytes compared with the unmodified polysaccharide, which is likely via their cross-linking and activation of this HA receptor (25, 52). HC1 has been directly implicated in HC·HA-mediated adhesion because its cleavage by the blood coagulation protease thrombin decreases the binding of monocytic cells to pathogenic HC·HA complexes, *i.e.* “HA cables” made by mucosal smooth muscle cells stimulated with a viral mimic (53). Interestingly, the proteolytic cleavage site on HC1 (^637^LGPRRTF^643^) overlaps with a flexible loop near the C-terminal end of HC1, where the R–R scissile bond is surface-exposed in our structure, such that digestion would release the globular portion of the heavy chain. In this regard, the free HC1 fragment could potentially act as a competitive inhibitor for interactions between HC·HA complexes, as well as reducing the number of cross-linking HC1–HC1 interactions.

We found that rHC1 was able to bind to complement C3 with moderate affinity (*K_D_* = ∼360 nm; Table 3), thereby identifying this complement component as a novel HC1 ligand. Modeling of the rHC1–C3 complex (Fig. 5) demonstrated that this interaction could be mediated via the C terminus of C3 co-chelating the metal ion within the MIDAS of HC1; *i.e.* consistent with the SPR data (Table 3). This also provides a plausible mechanism by which rHC1 inhibits the activity of the alternative pathway C3 convertase (Fig. 5*C*), by acting as a competitor of the interaction between FB and C3. Displacement of FB may also explain how IαI inhibits the factor D-mediated cleavage of FB to Bb (33), because this reaction requires FB to be associated with C3. In our functional assays, rHC1 was an ∼10-fold weaker inhibitor compared with FH, the only established negative regulator of the alternative pathway in the solution phase (45). Although IαI and FH have similar concentrations in serum, in tissues where HC1 accumulates via covalent attachment to HA, its complement inhibitory activity could serve to dampen the innate immune response, *e.g.* in complement-induced lung injury (29). Furthermore, HC1-mediated inhibition of complement activation might be particularly important during ovulation, where plasma proteins (including complement components and IαI) ingress into the ovarian follicle when the blood–follicle barrier breaks down. The presence of HC1 covalently associated with the cumulus matrix could provide protection to the COC from complement attack prior to its release into the oviduct.

The discovery that the vWFA of HC1 shares high structural similarity with vWFA domains of TEM8 and CMG2 may be significant given that these proteins are known to be functional receptors for the anthrax toxin (37), especially because IαI has been shown to protect against anthrax intoxication (54, 55). The latter has previously been attributed to the activity of the bikunin chain of IαI in inhibiting furin/proprotein convertases, which are proteases that have a critical role in the assembly of the anthrax toxin protective antigen (56). The protective antigen binds to the host cell surface by utilizing the receptors CMG2 and TEM8 (37), both of which contain vWFA domains that mediate the interaction via their MIDAS motifs (35, 36), in a similar manner to how integrins interact with their ligands. Thus, our structural data are consistent with a mechanism whereby IαI and HCs act as decoy receptors for the anthrax toxin and sequester the toxin in the fluid phase, preventing it from binding to membranes and forming the pores that give rise to the toxin's cytotoxic activity.

We have identified that rHC1 binds to vitronectin (a ligand of α_V_β_3_ and α_V_β_6_ integrins) with very high affinity (*K_D_* < 0.2 nm; Table 3), consistent with a previous report (31). However, our data clearly demonstrate for HC1 (at least) that the interaction with vitronectin does not involve the vWFA domain and is thus not a typical RGD-mediated MIDAS co-chelation interaction; *e.g.* an rHC1 construct lacking the entire vWFA domain bound to vitronectin with similar affinity to the WT protein (Fig. S6*C* and Table 3). The observation that the binding of IαI to vitronectin is inhibited by RGD peptides (31) is intriguing and suggests that even though this interaction is not mediated by metal ions, the integrin-binding site in vitronectin may be involved. However, our finding that the interaction of rHC1 with vitronectin is not competed by an RGD peptide argues against this. Further work is needed to localize the site on vitronectin that mediates binding to HC1 and determine the effect of HC1 on integrin–vitronectin interactions.

In light of the tight but noncanonical interaction of rHC1 with vitronectin and given that TGFβ1 and TGFβ3 interact with α_V_β_6_ via RGD sequences within their latency-associated peptides (47, 48), we screened the three small latent complexes of TGFβ for binding to rHC1. All three SLCs interacted with rHC1 with high affinity (*K_D_* = ∼10 nm; Table 3), including TGFβ2-LAP, which does not have an RGD motif. As in the case of vitronectin, the D298A mutant of rHC1 (with a defective MIDAS) bound the SLCs with similar affinities to WT rHC1. Additional SPR data indicated that rHC1 binds to the LAP rather than the mature growth factors and also interacts with the N-terminal region of LTBP1, which associates with TGFβ–LAP to form the LLC. Given that the LLCs sequester TGFβs in the matrix (50) through interactions with both the N- and C-terminal regions of LTBP1, it seems reasonable to suggest that HC1 may play a role in regulating the bioavailability of these important growth factors/cytokines. In this regard, whether HC1 acts in an analogous fashion to α_V_β6, *i.e.* to mechanically activate the release of mature TGFβ (57), or whether it stabilizes the LLC remains to be determined. The latter seems more likely based on its binding to both LAP and LTBP1 and is consistent with the finding that HC·HA complexes present in the human amniotic membrane, which may only contain HC1 (27), have been found to be potently tissue protective with anti-fibrotic activity (28).

Fibronectin, which binds to the α_5_β_1_ and α_V_β_3_ integrins, was found to interact with HC1 via its type III 13–14 repeats, a region of fibronectin that is well-characterized for its interaction with heparin. The binding of rHC1 to our cFN13–14 construct was of high affinity (*K_D_* = ∼15 nm; Table 3) but weaker than that with vitronectin and the TGFβ–LAP proteins. Not surprisingly, given that cFN13–14 does not contain an RGD sequence, the binding to rHC1 was metal ion– and MIDAS-independent and like vitronectin did not involve the vWFA domain. A lower affinity-binding site for rHC1 was also identified in the cell-binding region of fibronectin, and preliminary SPR studies (with the 50-kDa fragment) indicate a *K_D_* value for the interaction in the low micromolar range (data not shown).^4^

Further work will now be required to determine the molecular details for these noncanonical interactions of HC1 with integrin ligands and characterize their functional consequences. Moreover, whether the other ITI heavy chains interact with the ligands identified for HC1 needs to be established.

In summary, this study has identified that HC1 has a structural organization reminiscent of an integrin β-chain, including vWFA/hybrid domains and a functional MIDAS motif that mediates some but not all of its ligand-binding interactions. Our novel findings that HC1 can inhibit the complement alternative pathway by binding to C3 and has the potential to modulate TGFβ activity indicate that this protein is likely to be an important regulator of the innate and adaptive immune systems, for example, when it becomes covalently associated in the extracellular matrix during inflammation.

## Experimental procedures

### Protein production

The WT and D298A rHC1 proteins (amino acids 35–672 of ITH1 (isoform A) in UniProt) and a ΔvWFA mutant, lacking residues 288–478, were expressed and purified as described previously (58); all constructs contain a His6 tag (AHHHHHHVGTGSNDDDDKSPDP) N-terminal to the rHC1 sequence. The cFN13–14 protein was expressed in *E. coli.* A codon-optimized gene, encoding the N-terminal sequence MCHHHHHHDDDK followed by amino acid residues 1812–1991 of human fibronectin (Uniprot: P02751), was cloned into pET-11a, using NdeI and BamHI restriction sites, by Genscript Inc. Transformed BL21(DE3) pLysS cells (Promega) were cultured in Terrific broth at 37 °C, and protein expression was induced by addition of isopropyl β-d-thiogalactopyranoside (0.2 mm final concentration) at an OD_600 nm_ between 0.4 and 0.6, followed by incubation at 30 °C for 12 h. Harvested cells were lysed using BugBuster (primary amine free) protein extraction reagent (Novogen), according to the manufacturer's instructions. The cFN13–14 was captured from the clarified supernatant on nickel Sepharose 6 Fast Flow beads (GE Life Sciences), followed by purification on a HisTrap HP column (GE Life Sciences) and then separation of monomeric and dimeric cFN13–14 by affinity chromatography on a HiTrap heparin HP column (GE Life Sciences); the latter is shown on Fig. S7. The monomeric protein was found to be pure by SDS-PAGE (Fig. S7), and electrospray ionization MS demonstrated that it corresponded to a molecular mass within 1 Da of the theoretical value for the construct with the N-terminal methionine removed (21,264.3 and 21,265.0 Da, respectively). One-dimensional NMR spectroscopy gave a spectrum consistent with that of a folded protein, *i.e.* with high-field shifted methyl resonances and good dispersion in the amide region (data not shown).

The recombinant domains of LTBP1 (NT1, EGF, and CT) were produced as described before (59). LAP (from TGFβ1), TGFβ1, TGFβ1-LAP, TGFβ2-LAP, TGFβ3, and TGFβ3-LAP were obtained from R&D Systems, vitronectin was from PeproTech, and complement C3 was from Merck. IαI was purified from human plasma as described previously (1).

### Crystal structure determination of rHC1

Following the observation of metal ion-dependent dimerization of rHC1 in initial SAXS studies, we conducted crystallization screens using the D298A mutant that is monomeric. Conditions that led to crystals for D298A were then used for the WT protein. WT rHC1 and the D298A mutant were crystallized by mixing 1 μl of protein (10 mg/ml in 10 mm HEPES, pH 7.5, 50 mm NaCl) with an equal amount of crystallization mother liquor (100 mm HEPES, pH 7.5, 100 mm sodium acetate, 10% (w/v) PEG8K, 20% (v/v) glycerol). Crystals appeared within 1 week. Native diffraction data were collected to 2.20 Å (D298A) and 2.34 Å (WT), and the data were indexed, integrated, and scaled using DIALS (60), POINTLESS (61), AIMLESS/SCALA (62, 63), and cTRUNCATE (61) as implemented in the Xia2 pipeline (64). The data were phased using the SIRAS method and a K_2_PtCl_4_-derivatised D298A crystal. The substructure was solved, and the data was phased, density was modified, and the chain was partially traced using PHENIX AutoSol (65). Both the WT and D298A models were rebuilt and refined to convergence using the COOT (66) and PHENIX Refine (67) packages. The asymmetric unit of the crystals for both WT and D298A contained two independent copies of rHC1; data collection statistics are shown in Table 1. The refined models have been deposited in the PDB with accession codes 6FPY (WT) and 6PFZ (D298A).

### Small angle X-ray scattering and modeling of IαI, rHC1, and rHC1–C3 complex

SAXS data were collected at Beamline P12, PetraIII, DESY (68). Proteins (rHC1 or IαI) at 1.25, 2.5, and 5.0 mg/ml were prepared in HEPES-buffered saline (pH 7.5). The data were reduced using PRIMUS/GNOM (69, 70). The *R*_g_ and *D*_max_ values shown in Table 2 were calculated automatically using AUTORG and DATGNOM (71) to prevent bias or subjective interpretation. *Ab initio* models were created using the DAMMIF/DAMMIN packages (72, 73); 20 models were made using DAMMIF in slow mode. The averaged model from DAMMIF was refined to convergence using DAMMIN. Modeling of residues missing from the crystal structure was done using the AllosMod-FoXs server (74). Modeling of the dimeric form of HC1 was carried out as for the monomeric form, although P2 symmetry was enforced once it was determined that the data corresponded to a dimer. Rigid body docking of the HC1 structure into the resulting DAMMIN envelope was performed in UCSF Chimera for both monomeric and dimeric HC1. The resolution of the resulting map used for fitting was determined using SASRES (75); this was 43 Å for the monomer and 64 Å for the dimer. In modeling of the dimer, we enforced the 2-fold axis from the DAMMIN model. A threading model of HC2 was generated from the structure of HC1 using Phyre (76) and modeled along with bikunin (44) and HC1 into the DAMMIN envelope using Sculptor (77) simultaneous docking protocols.

Structures of the rHC1 vWFA domain (this study) and the complement C3 C-terminal C345C domain (PDB 2XWJ (46)) were positioned relative to each other informed by the C3-FB complex (PDB 2XWJ). The models were locally docked to each other using Rosetta_3.2 and the standard docking protocol (78), with random perturbations of 3 Å and 8°; 10,000 models were generated, and the lowest energy model is shown in Fig. 5*D*. Additionally, a SAXS envelope was generated of full-length C3 bound to rHC1 in the presence of 2 mm MnCl_2_. DAMMIN envelopes were calculated as described above. Crystal structures of C3 (PDB 2A73 (79)) and HC1 (this study) were docked into this envelope using Sculptor (77).

### Analytical ultracentrifugation of rHC1

The metal ion dependence of rHC1 dimerization was analyzed using both velocity and equilibrium AUC. All AUC experiments were conducted at 20 °C on a Beckman XL-A ultracentrifuge with an An60Ti rotor.

For velocity AUC, 18 μm WT rHC1 protein was prepared in HEPES-buffered saline, pH 7.5, in the presence of either 2.5 mm EDTA or 5 mm MgCl_2_. The samples were analyzed at 40,000 rpm for 5 h, with scans taken at 280 nm every 90 s. This experiment was conducted in triplicate with representative data shown in Fig. 3*A*. Sedimentation coefficient distributions (*c*(*s*)) were calculated using SEDFIT (80).

For equilibrium AUC, measurements were made at three different concentrations of rHC1 (4, 11, and 22 μm), and each of these were prepared with five different concentrations of MgCl_2_ (0 (2.5 mm EDTA), 0.1, 0.5, 1, and 5 mm). Rotor speeds of 10,000, 15,000, and 20,000 rpm were used with scans at 280 nm (and 290 nm for the highest concentration) after equilibrium had been reached (18 h). The data (from triplicate experiments) were analyzed by global analysis with SEDFIT/SEDPHAT (81) and fitted to a monomer–dimer model.

### Surface plasmon resonance of rHC1–ligand interactions

SPR experiments were conducted on either a Biacore 3000 or T200 instrument. For C3 binding assays, ∼2,000 RU of C3 was immobilized on a CM5 chip using standard amide coupling chemistry, and rHC1 was injected at a range of concentrations (1–31.25 μm) over the chip surface. For all other assays, 1,000 RU (for cFN13–14) or 1,500 RU of rHC1 proteins (WT, D298A or ΔvWFA) were immobilized on a C1 chip by amide cross-linking chemistry, and cFN13–14 (7.5–120 nm), LAP (from TGFβ1; 3.125–200 nm), LTBP1 (NT1, EGF, or CT domains; all at 0.156–10 nm), TGFβ1, TGFβ3 (both at 7.8–500 nm), TGFβ1-LAP, TGFβ2-LAP, TGFβ3-LAP (all at 3.125–200 nm), or vitronectin (0.3125–10 nm) was used as the analyte.

Except for cFN13–14, experiments were conducted in HEPES-buffered saline, pH 7.5, with 0.05% (v/v) Tween 20. Metal ions (2 mm) or chelating agent (EDTA; 10 mm) were added to the buffers, and a flow rate of 50 μl/min was used when generating kinetic parameters. The data were collected in triplicate, and *K_D_* values (means ± S.D. in Table 3) were determined from multicycle kinetics.

For cFN13–14, *K_D_* values were determined from single cycle kinetics (three independent experiments) where cFN13–14 was flowed over the immobilized rHC1 proteins at 7.5, 15, 30, 60, and 120 nm (in HEPES-buffered saline, pH 7.4, 0.05% Tween 20) for 60 s each at a flow rate of 30 μl/min.

The data were fitted to a Langmuir 1:1 model using the BIAeval T200 software. For all fits, the χ^2^ value obtained was less than 10% of the *R*_max_ value.

### C3 convertase assay

Inhibition of C3 activation to C3b was measured using a fluid phase convertase assay. Here C3 (19.5 μm) was incubated with 1.75 μm complement FB and 0.37 μm complement factor D in 20 mm HEPES, 130 mm NaCl, 3 mm MgCl_2_, 1 mm EGTA, pH 7.5. The effect of rHC1 (preincubated with 1 mm MnCl_2_) was measured at concentrations ranging from 0 to 27 μm; complement FH was used as a positive control. After 1-min incubation at 37 °C, the reaction was stopped by addition of 5× SDS loading buffer, and the samples were incubated at 100 °C for 5 min. The samples were run on a 4–12% gradient SDS-PAGE gel and stained with Coomassie Blue. C3a formation was monitored by densitometry using an Odyssey imaging system (LI-COR Biosciences)

### Data availability

The structures of WT and D298A HC1 have been deposited at the Research Collaboratory for Structural Bioinformatics PBD with accession codes 6FPY and 6FPZ, respectively. AUC, SAXS, and SPR data will be shared upon request. All other data are contained within the manuscript.

## Author contributions

D. C. B. and A. J. D. conceptualization; D. C. B., C.M.K., J. J. E., C. B., C. M. M., and A. J. D. resources; D. C. B. data curation; D. C. B. and T. A. J. formal analysis; D. C. B., A. W. W. L.-S., and H. L. B. investigation; D. C. B. visualization; D. C. B., A. W. W. L.-S., H. L. B., and T. A. J. methodology; D. C. B. and A. J. D. writing-original draft; D. C. B., A. W. W. L.-S., H. L. B., T. A. J., C. M. K., J. J. E., C. B., C. M. M., and A. J. D. writing-review and editing; C. M. M. and A. J. D. supervision; C. M. M. and A. J. D. funding acquisition; A. J. D. project administration.

## Supplementary Material

Supporting Information

## Footnotes

4 D. C. Briggs, H. L. Birchenough, T. A. Jowitt, and A. J. Day, unpublished data.

## Footnotes

3 The abbreviations used are:

IαI: inter-α-inhibitor
AUC: analytical ultracentrifugation
CMG2: capillary morphogenesis protein-2
COC: cumulus–oocyte complex
CS: chondroitin sulfate
FB: complement factor B
HA: hyaluronan
HC: heavy chain
ITGB: integrin β-chain
LAP: latency associated peptide
LLC: large latent complex
LTBP: latent TGFβ-binding protein
MIDAS: metal ion-dependent adhesion site
PαI: pre-α-inhibitor
rHC1: recombinant HC1
SAXS: small-angle X-ray scattering
SLC: small latent complex
TEM8: tumor endothelial marker-8
TGFβ: transforming growth factor β
TSG-6: tumor necrosis factor–stimulated gene 6
vWFA: von Willebrand factor A
RMSD: root-mean-square deviation
PDB: Protein Data Bank
SPR: surface plasmon resonance
FH: factor H
NT1: N-terminal region
CT: C-terminal region
EGF: epidermal growth factor.
