# Supplementary material for: Inter-α-inhibitor heavy chain-1 has an integrin-like 3D structure mediating immune regulatory activities and matrix stabilization during ovulation
Source: J Biol Chem. 2020 Mar 6;295(16):5278–91. doi: 10.1074/jbc.RA119.011916 (PMC7170535; doi:10.1074/jbc.RA119.011916)
Supplement: Supporting Information [file supp_295_16_5278__index.html]

Inter-α-inhibitor heavy chain-1 has an integrin-like 3D structure mediating immune regulatory activities and matrix stabilization during ovulation — ITIH1 structure reveals roles in inflammation & ovulation — Inter-α-inhibitor heavy chain-1 has an integrin-like 3D structure mediating immune regulatory activities and matrix stabilization during ovulation — ITIH1 structure reveals roles in inflammation and ovulation — Supporting Information 

# Inter-α-inhibitor heavy chain-1 has an integrin-like 3D structure mediating immune regulatory activities and matrix stabilization during ovulation

## Supporting Information

- Supporting Information (to be published online) - Supporting Information - Figures and legends
