# Supplementary material for: Inter-α-inhibitor heavy chain-1 has an integrin-like 3D structure mediating immune regulatory activities and matrix stabilization during ovulation
Source: J Biol Chem. 2020 Mar 6;295(16):5278–91. doi: 10.1074/jbc.RA119.011916 (PMC7170535; doi:10.1074/jbc.RA119.011916)
Supplement: Supporting Information [file supp_RA119.011916_157151_1_supp_478206_q5yl13.pdf]

SUPPORTING INFORMATION

FIGURE LEGENDS

Figure S1: Schematics of IαI and PαI, TSG-6-mediated HC transfer and domain organizations for HCs 1-3. **A)** A schematic illustrating the organization of IαI and PαI showing that these proteoglycans both contain the bikunin core protein to which a chondroitin sulphate (CS) chain is attached via a typical tetrasaccharide linkage; heavy chains (HC1 and HC2 in IαI and HC3 in PαI) are linked to CS via ester bonds (red circles) formed between their C-terminal aspartic acid residues and a C6 hydroxyl within a N-acetyl galactosamine sugar in CS. In the presence of the transesterase TSG-6 and hyaluronan (HA), which is composed of a variable number (n) of repeating disaccharides of glucuronic acid (diamonds) and N-acetyl glucosamine (squares), HCs are covalently transferred from IαI/PαI onto HA to form HC•HA complexes; ester bonds link the C-terminal aspartic acids of the HCs to N-acetyl glucosamine residues of HA. **B)** A schematic of the domain organization of the mature HC1 protein (residues 35-672 in Uniprot P19827), as determined from the crystal structure described here, along with their corresponding structural elements. The N- and C-terminal regions associate to form the Hybrid2 domain and the vWFA domain is flanked by H-sequences that constitute the Hybrid1 domain; the position of the D298A mutant is indicated along with the region that is deleted in the ΔvWFA construct. The domain organizations of the mature HC2 and HC3 proteins can be inferred from their homology with HC1 (39% and 54% identity, respectively).

Figure S2: Equilibrium AUC on rHC1 in absence and presence of $MgCl_2$ ions. Three concentrations of rHC1 (4, 11 and 22 μM) were analysed by equilibrium AUC at rotor speeds of 10,000 (pink), 15,000 (blue) and 20,000 (cyan) rpm in the absence (2.5 mM EDTA) or presence of 0.1, 0.5, 1 or 5 mM $MgCl_2$. High speed data for 11 μM HC1 in 1 mM $MgCl_2$ were omitted.

Figure S3: SAXS data analysis for HC1 monomer (orange) and dimer (blue). A) Analysis of the Guinier region and residuals. **B)** Dimensionless Kratky plots show that HC1 monomer and dimer molecules are folded and globular; the cross-hairs denote the globularity point, and the shift of the maxima for the HC1 dimer to the right indicate that it is more extended and asymmetric than the monomer. SIBYLS (**C**) and Porod-Debye (**D**) plots indicate that the HC1 monomer and dimer are rigid.

Figure S4: **SAXS data analysis for IαI. A)** Analysis of the Guinier region and residuals. **B)** Dimensionless Kratky plots show that all of IαI is folded, globular, but asymmetric as revealed by the maxima being to the right of the globularity point (cross-hairs). SIBYLS (**C**) and Porod-Debye (**D**) plots demonstrate that IαI is rigid.

Figure S5: **SAXS data analysis for the rHC1-C3 complex. A)** Analysis of the Guinier region and residuals. **B)** A Dimensionless Kratky plot reveals that the rHC1-C3 complex is folded, globular, but asymmetric as revealed by the maxima being to the right of the globularity point (cross-hairs). SIBYLS (**C**) and Porod-Debye (**D**) plots indicate that the rHC1-C3 complex has some flexibility.

Figure S6: MIDAS-independent binding of HC1 to vitronectin and TGFβ-LAP proteins. SPR sensorgrams for the interaction of vitronectin (Vn), at concentrations of 0.625 nM, 1.25 nM, 2.5 nM, 5 nM and 10 nM with immobilized WT **(A)**, D298A **(B)** or ΔvWFa **(C)** rHC1. Data are representative of 3 independent experiments with derived numerical values shown in Table 3. **D)** Representative SPR sensorgrams for the binding of TGFβ-LAP proteins (TGFβ1-LAP (green); TGFβ2-LAP (orange or red); TGFβ3-LAP (purple or blue)) with immobilised rHC1 (WT (light green, orange, purple) or D298A (dark green, red, blue)). The individual interactions were analyzed further in 3 independent experiments (using different concentrations of TGFβ-LAP proteins) to generate the data in Table 3.

Figure S7: Heparin affinity purification of cFN13-14. A) The final stage of purification for cFN13-14 was on a 5-ml HiTrap Heparin HP equilibrated in 20 mM Tris, 150 mM NaCl, 2 mM EDTA, pH 7.3. The protein was eluted with a gradient of 0.15 M – 1 M NaCl (in equilibration buffer) at a flow rate of 5 ml/min over 20 column volumes, with absorbance monitored at 280 nm. Two peaks eluted (P1 and P2) and these were analyzed by SDS-PAGE under **(B)** reducing (R) or **(C)** non-reducing (NR) conditions. Peak P1 (~22 kDa) corresponded to monomeric cFN13-14, whereas P2 was predominantly dimeric (~40 kDa). Fractions from P1 corresponding to cFN13-14 monomer were pooled and dialyzed against HEPES buffered saline, pH 7.4.

Figure S8: MIDAS-independent binding of HC1 to cFN13-14. SPR sensorgrams (from single cycle kinetics) for the interaction of cFN13-14, at concentrations of 7.5 nM, 15 nM, 30 nM, 60 nM and 120 nM, with immobilized WT **(A)**, D298A **(B)** or ΔvWFa **(C)** rHC1. Data are representative of 3 independent experiments, with derived numerical values shown in Table 3.

Figure S1

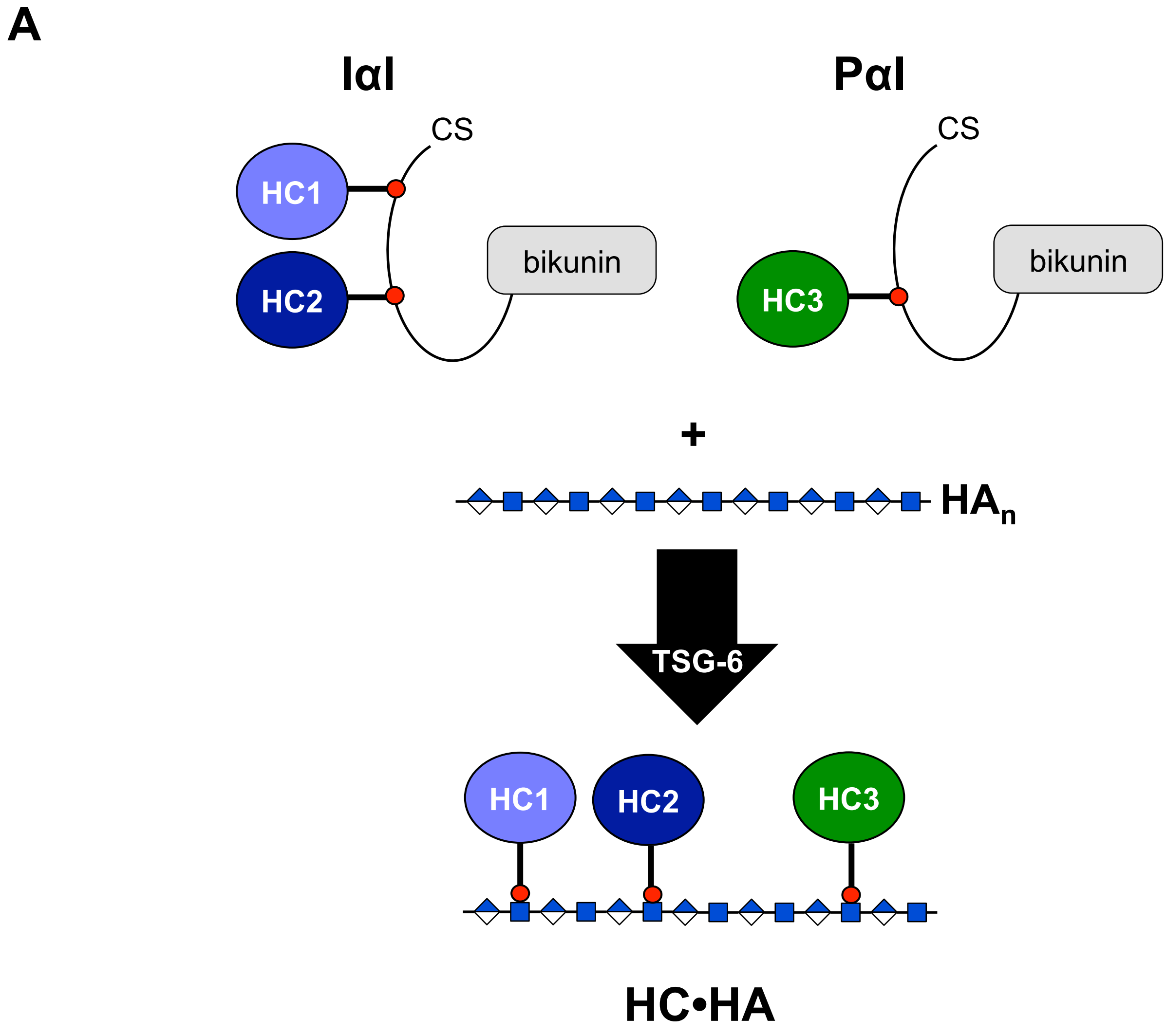
A
IαI
PαI
CS
CS
HC1
HC2
HC3
bikunin
bikunin
+
HAn
TSG-6
HC1
HC2
HC3
HC•HA

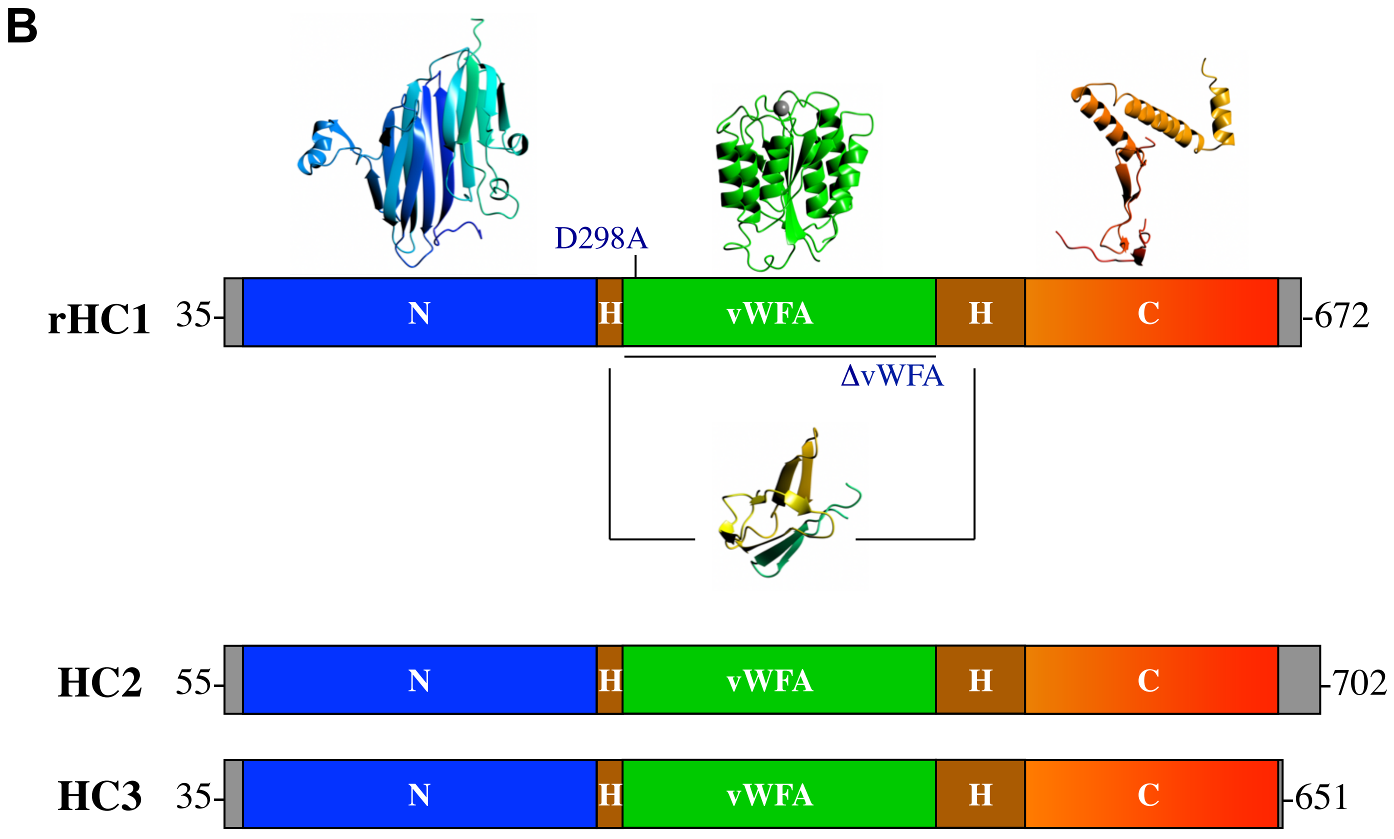
B
D298A
rHC1
35-
N
H
vWFA
H
C
-672
ΔvWFA
HC2
55-
N
H
vWFA
H
C
-702
HC3
35-
N
H
vWFA
H
C
-651

Figure S2

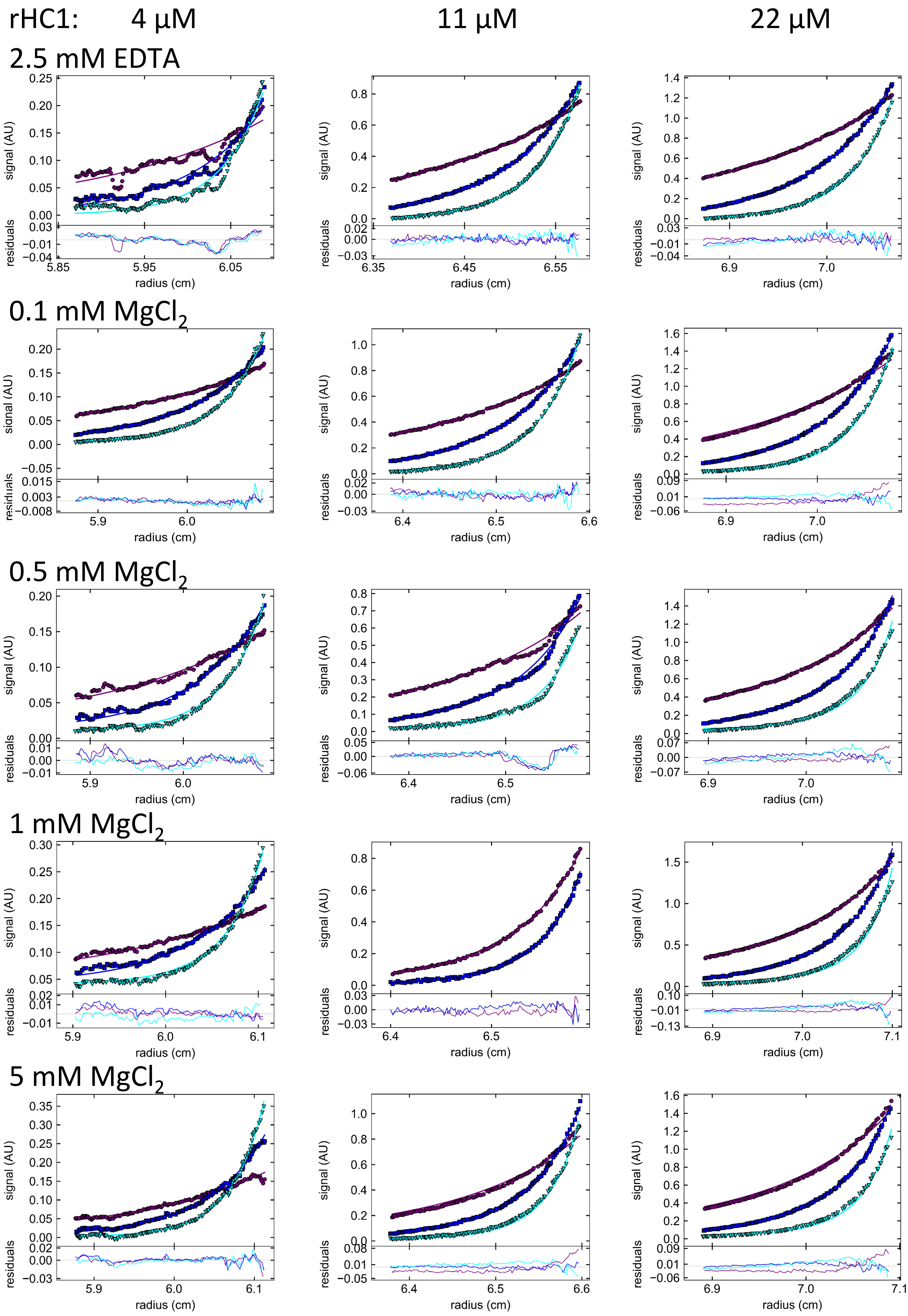

rHC1:
4 µM
11 µM
22 µM
2.5 mM EDTA
0.1 mM MgCl2
0.5 mM MgCl2
1 mM MgCl2
5 mM MgCl2
signal (AU)
residuals
radius (cm)

Figure S3

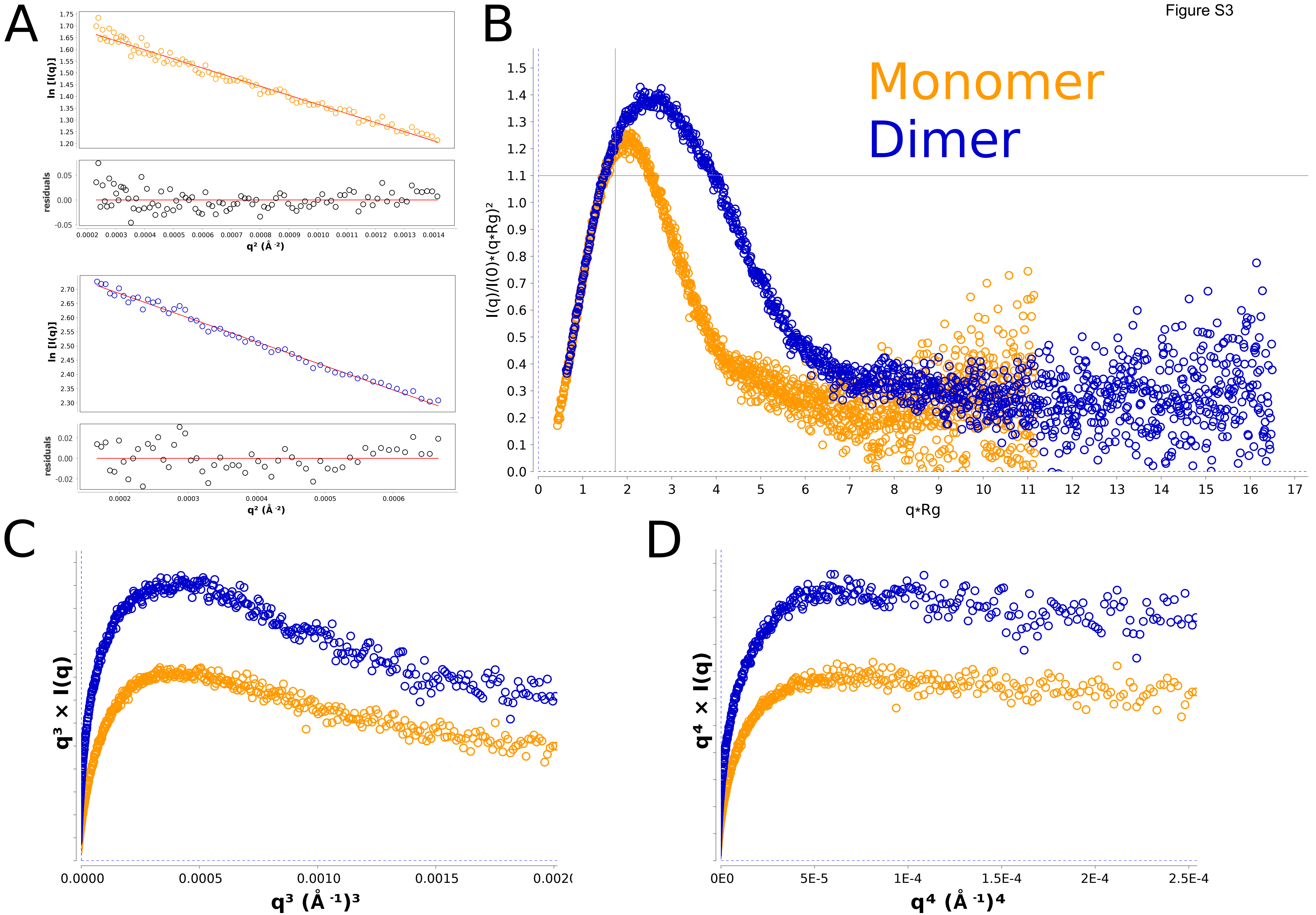
A
ln [I(q)]
residuals
q² (Å⁻²)
B
Monomer
Dimer
I(q)/I(0)*(q*Rg)²
q*Rg
C
q³ × I(q)
q³ (Å⁻¹)³
D
q⁴ × I(q)
q⁴ (Å⁻¹)⁴

Figure S4

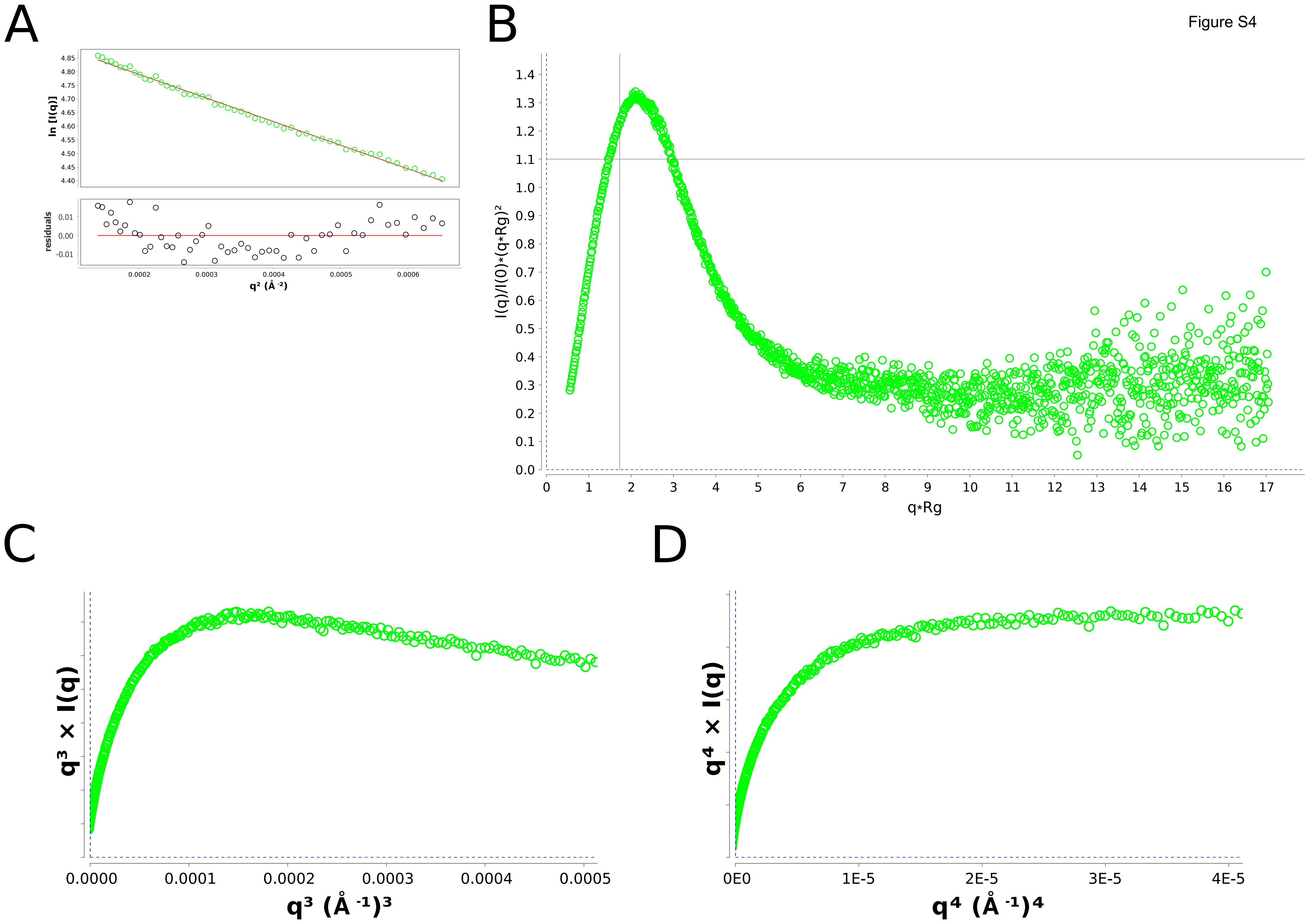
A
ln [I(q)]
4.85
4.80
4.75
4.70
4.65
4.60
4.55
4.50
4.45
4.40
residuals
0.01
0.00
-0.01
0.0002
0.0003
0.0004
0.0005
0.0006
q² (Å ⁻²)
B
I(q)/I(0)*(q*Rg)²
1.4
1.3
1.2
1.1
1.0
0.9
0.8
0.7
0.6
0.5
0.4
0.3
0.2
0.1
0.0
0 1 2 3 4 5 6 7 8 9 10 11 12 13 14 15 16 17
q*Rg
C
q³ × I(q)
0.0000
0.0001
0.0002
0.0003
0.0004
0.0005
q³ (Å ⁻¹)³
D
q⁴ × I(q)
0E0
1E-5
2E-5
3E-5
4E-5
q⁴ (Å ⁻¹)⁴

Figure S5

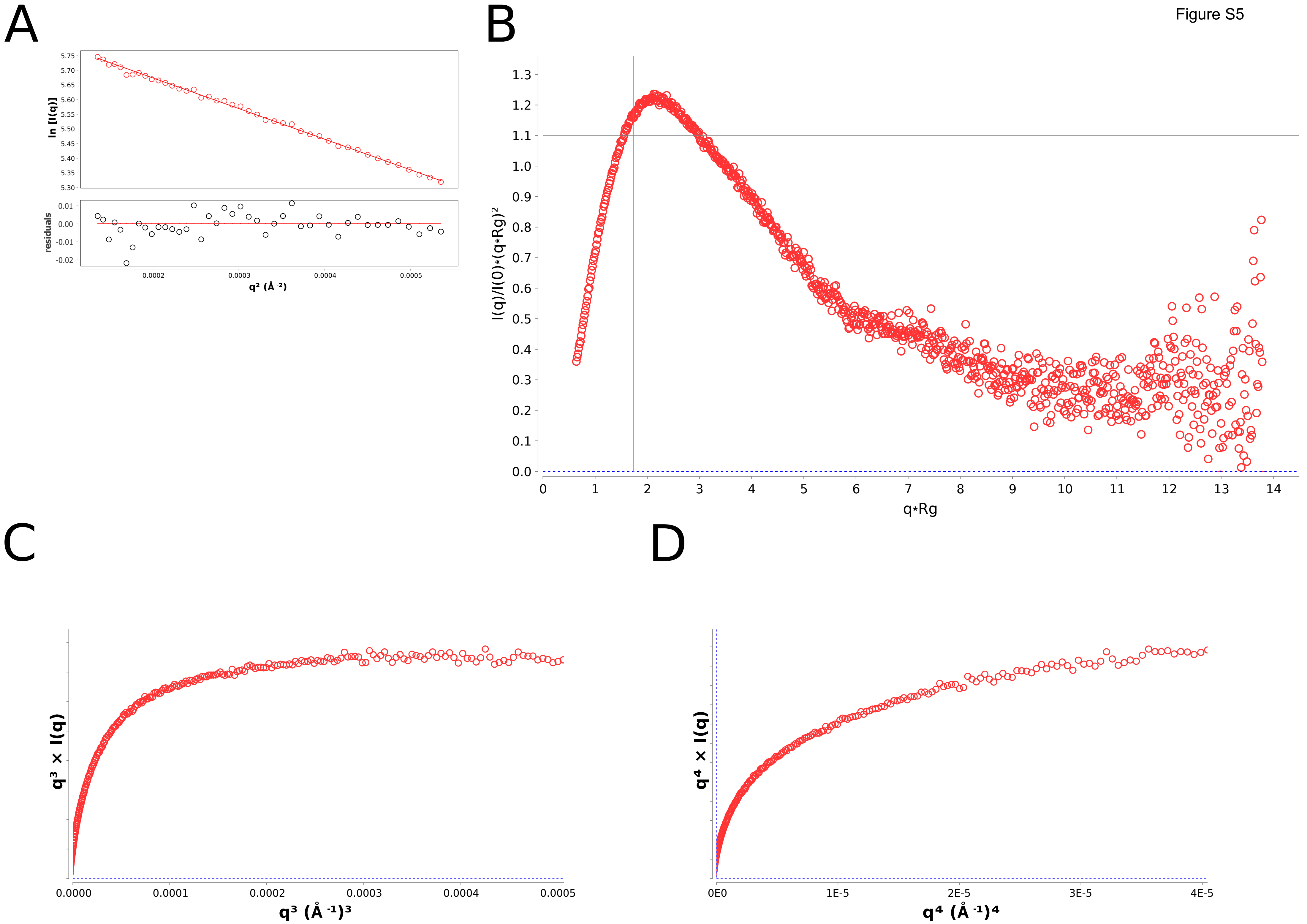
A
ln [I(q)]
residuals
q² (Å ⁻²)
B
I(q)/I(0)*(q*Rg)²
q*Rg
C
q³ × I(q)
q³ (Å ⁻¹)³
D
q⁴ × I(q)
q⁴ (Å ⁻¹)⁴

Figure S6

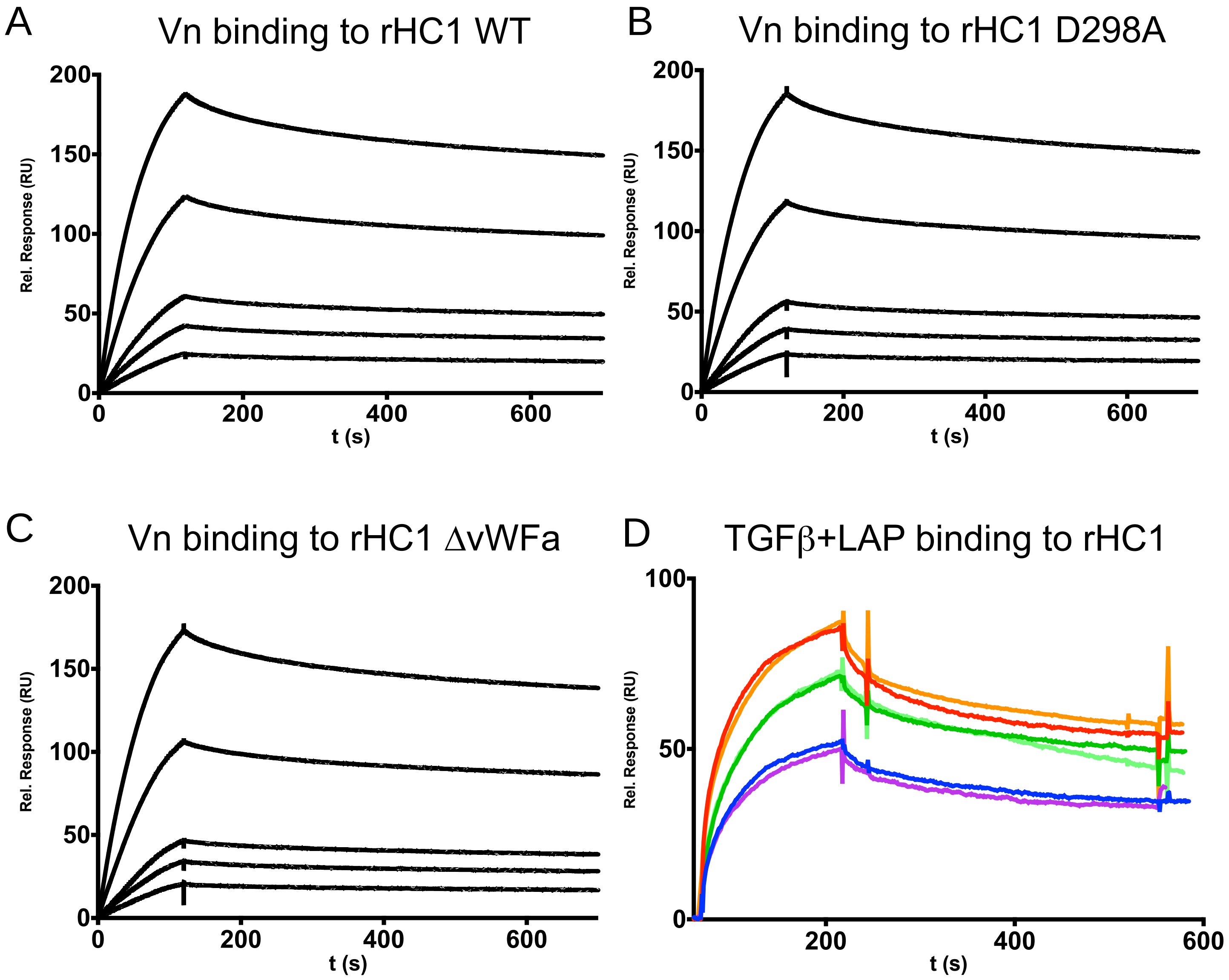
A
Vn binding to rHC1 WT
B
Vn binding to rHC1 D298A
C
Vn binding to rHC1 ΔvWFa
D
TGFβ+LAP binding to rHC1
Rel. Response (RU)
t (s)
0
50
100
150
200
400
600

Figure S7

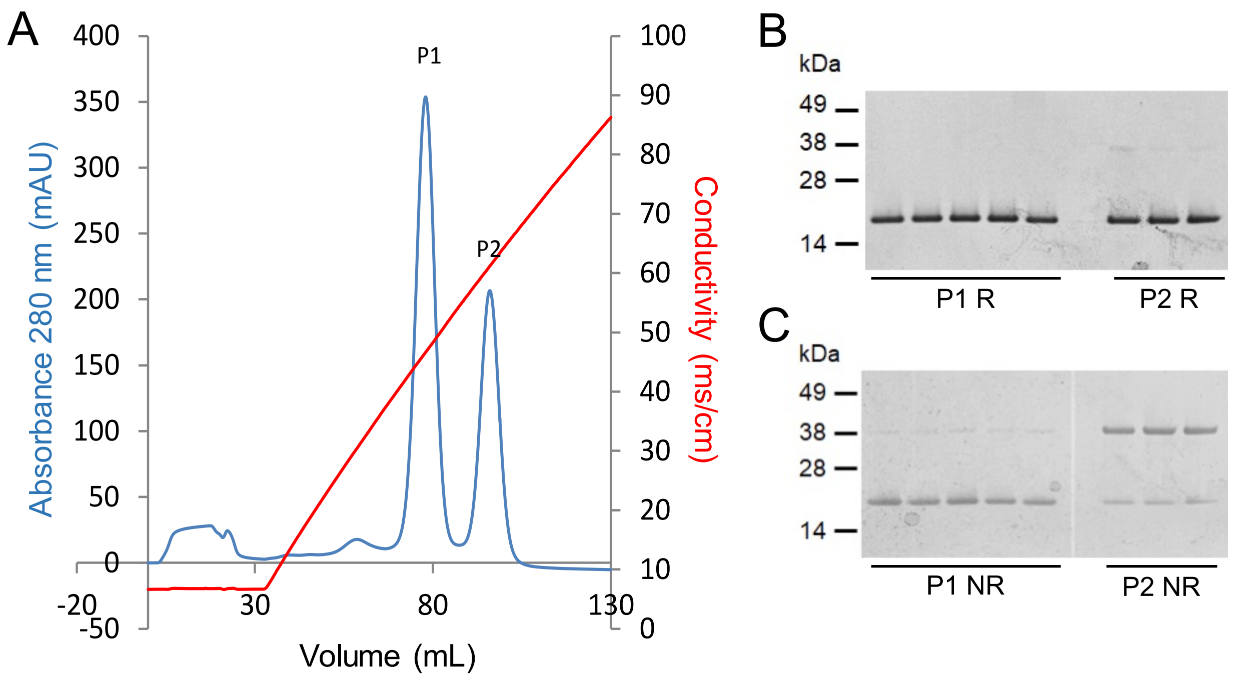
A
B
C
P1
P2
Absorbance 280 nm (mAU)
Conductivity (ms/cm)
Volume (mL)
400
350
300
250
200
150
100
50
0
-50
-20
30
80
130
100
90
80
70
60
50
40
30
20
10
0
kDa
49
38
28
14
P1 R
P2 R
P1 NR
P2 NR

Figure S8

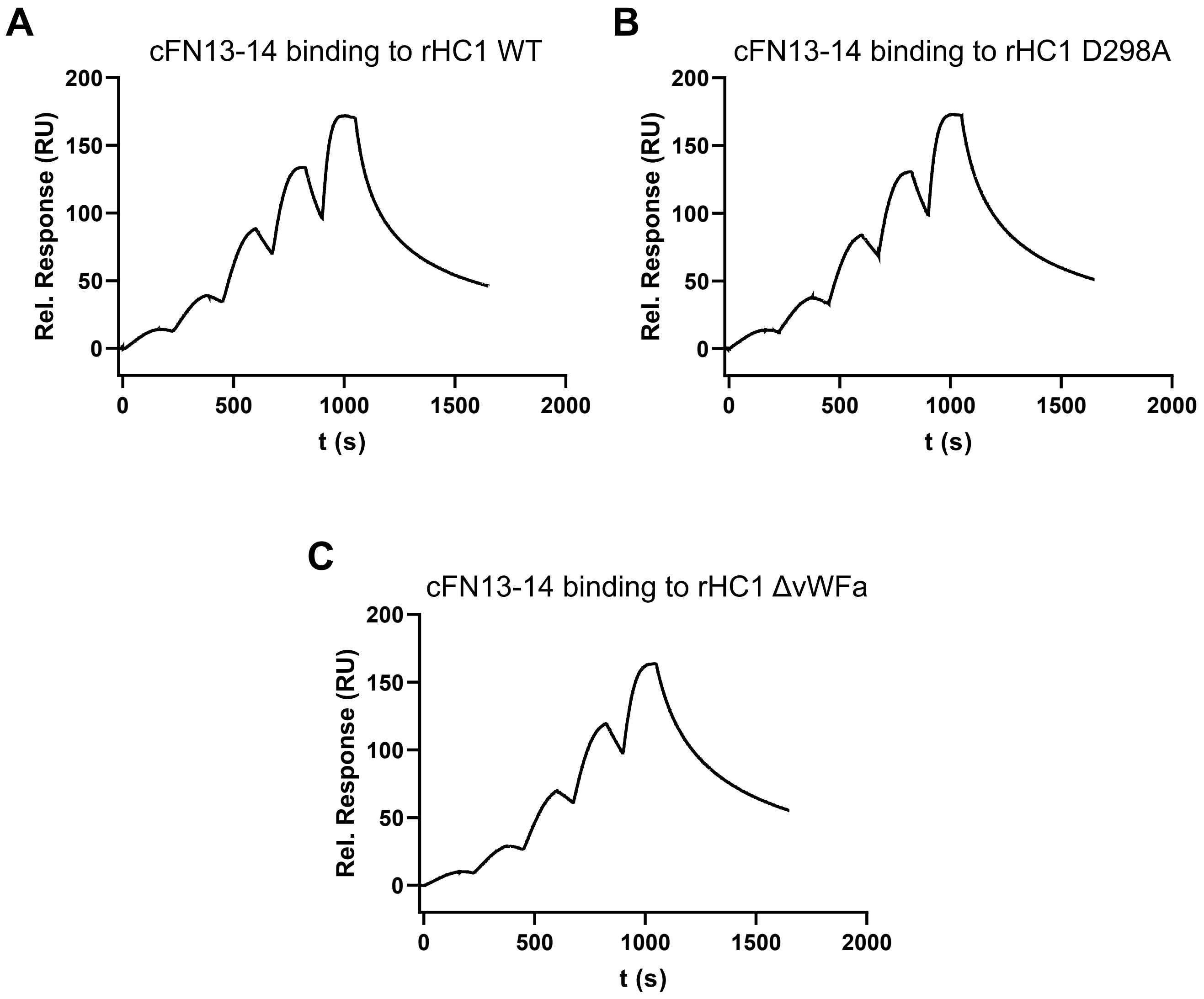
A
cFN13-14 binding to rHC1 WT
Rel. Response (RU)
0
50
100
150
200
t (s)
0
500
1000
1500
2000
B
cFN13-14 binding to rHC1 D298A
Rel. Response (RU)
0
50
100
150
200
t (s)
0
500
1000
1500
2000
C
cFN13-14 binding to rHC1 ΔvWFa
Rel. Response (RU)
0
50
100
150
200
t (s)
0
500
1000
1500
2000
